# Salicylic acid inhibits V-ATPase activity and restricts cell elongation

**DOI:** 10.1093/plphys/kiaf439

**Published:** 2025-09-26

**Authors:** Jonas Müller, Yvonne König, Kaltra Xhelilaj, Sabrina Kaiser, Tobias Müller, Alejandra Vielba-Fernández, Julien Gronnier, Christian Löfke, Melanie Krebs, David Scheuring

**Affiliations:** Department of Biology, Plant Pathology, University of Kaiserslautern-Landau, Kaiserslautern 67663, Germany; Department of Biology, Plant Pathology, University of Kaiserslautern-Landau, Kaiserslautern 67663, Germany; Center for Plant Molecular Biology (ZMBP), University of Tübingen, Tübingen 72076, Germany; Department of Biology, Plant Pathology, University of Kaiserslautern-Landau, Kaiserslautern 67663, Germany; Department of Biology, Plant Pathology, University of Kaiserslautern-Landau, Kaiserslautern 67663, Germany; Department of Biology, Plant Pathology, University of Kaiserslautern-Landau, Kaiserslautern 67663, Germany; Center for Plant Molecular Biology (ZMBP), University of Tübingen, Tübingen 72076, Germany; Plant Cell Biology, School of Life Sciences, Technical University of Munich (TUM), Freising 85354, Germany; BioBloom, Apetlon 7143, Austria; Cell Biology, Centre for Organismal Studies (COS), Heidelberg University, Heidelberg 69120, Germany; Department of Biology, Plant Pathology, University of Kaiserslautern-Landau, Kaiserslautern 67663, Germany

## Abstract

While the role of salicylic acid (SA) in plant defence has been investigated for decades, its role in regulating plant growth and development has only come into focus recently. SA application inhibits growth independently of the established “Nonexpressor of Pathogenesis Related” (NPR) receptors. However, the underlying mechanism at the cellular level remains largely elusive. Here, we show that SA induces changes in vacuolar morphology and a significant increase in vacuolar pH in Arabidopsis (*Arabidopsis thaliana*). We demonstrate SA-mediated inhibition of V-ATPase activity, which is confirmed by experiments using the V-ATPase mutant *vha-a2 vha-a3*. The observed effects seem to be independent of the phytohormone auxin, which has been reported to crosstalk with SA. By inhibiting V-ATPase activity, SA impacts basic cellular functions such as vesicle trafficking and/or nutrient storage, affecting cell size and growth. Our results reveal a NPR-independent mechanism that attenuates growth, potentially reallocating resources to enhance plant robustness and promote endurance during environmental stresses.

## Teaser

The phytohormone salicylic acid directly inhibits V-ATPase activity and cell elongation thereby limiting plant growth.

## Introduction

Plants have evolved efficient measures to oppose environmental stresses. SA is one of the key molecules for stress signaling, e.g. in response to pathogen attacks. A rise of endogenous SA levels induces efficient local and systemic defence responses as a countermeasure (Loake and Grant 2007). This comes, however, to the expense of reduced or even inhibited growth. In the last decade, the role of SA in plant defence has been well investigated but the inhibitory effect on plant growth is considerably less understood. Recently, SA function during plant growth and development came into focus (Rivas-San Vicente and Plasencia 2011). Notably, the signaling cascade mediating SA-induced growth attenuation seems to be independent of its defence-inducing role. It was demonstrated that the SA receptor Nonexpressor of Pathogenesis Related genes 1 (NPR1), the key regulator of plant immune responses, is not responsible for the transmission of growth-restriction when exogenously applied (Tan et al. 2020).

While long distance transport of methylated SA via the phloem to distal tissues to induce systemic acquired resistance (SAR) has been well established (Park et al. 2007), only one intracellular SA transporter has been identified to date (Anfang and Shani 2021). The MATE family transporter EDS5 was demonstrated to be crucial for SA export from the chloroplast, where it is synthesized, to the cytosol (Serrano et al. 2013). There is, however, biochemical evidence that glycosylated SA is transported into the vacuole for inactivation (Bonnemain et al. 2013). Vacuolar transport of glycosylated SA was shown to require either an ATP-binding cassette transporter mechanism or an H^+^-antiport mechanism, dependent on the investigated plant species (Dean and Mills 2004; Dean et al. 2005). The glycosylation step prior to vacuole uptake in Arabidopsis is dependent on the small-molecule glucosyltransferases UGT74F1, UGT74F2, and UGT76B1. The latter seems to be central for controlling levels of free and active SA, thereby contributing to balance the plant immune status (von Saint Paul et al. 2011; Bauer et al. 2021).

Recently, crosstalk of SA with the phytohormone auxin was demonstrated (Pasternak et al. 2019). SA impacted auxin synthesis and transport which eventually led to changes in root architecture. Pasternak et al. (2019) showed that low SA concentrations promoted adventitious root emergence and changed the organization of the root meristem, while high SA concentrations (above 250 *µ*M) led to general inhibition of root growth. More recently, also lower SA concentrations were found to attenuate root growth, and the reported SA-auxin crosstalk was mechanistically explained by SA binding to A subunits of protein phosphatase 2A (PP2A), leading to inhibition of its function and ultimately to changes of the PIN2 auxin transporter (Tan et al. 2020). SA-induced PP2A inhibition causes hyperphosphorylation of PIN2 and loss of its cellular polarity, severely affecting root growth and development. In line with this, endogenous and exogenously applied SA inhibits clathrin-mediated endocytosis and thus affects internalization of plasma membrane (PM) proteins such as PINs (Du et al. 2013). However, since PP2A acts on several substrates and *pp2aa1* mutation led to increased SA sensitivity (Tan et al. 2020), it seems likely that other molecular players are involved.

In the past, it was shown that auxin has an inhibiting effect on underground tissues (Evans et al. 1994). Interestingly, auxin has been shown to impact the cells largest organelle—the vacuole, leading to smaller vacuoles and restricted cell elongation (Löfke et al. 2015a; Scheuring et al. 2016). In this context, a *space-filling* function was defined for the vacuole: simple inflation and occupation of emerging cellular space allows for rapid cell elongation. Thus, energy consumption for the generation of cytosolic content can be reduced and efficient growth maintained (Scheuring et al. 2016; Krüger and Schumacher 2018; Dünser et al. 2019; Kaiser and Scheuring 2020). The auxin-induced reduction in vacuole size is dependent on the actin cytoskeleton (Scheuring et al. 2016; Kaiser et al. 2019), and endocytic trafficking has been demonstrated to promote vacuole enlargement (Dünser et al. 2022). Cell elongation and eventually root growth in turn seems to depend on unconditioned vacuole inflation, leading to the hypothesis that reduction of vacuole size might be a general mechanism to reduce and control growth (Kaiser et al. 2021).

Here, we show that cell size is restricted by application of moderate SA concentrations. This is accompanied by changes of vacuolar morphology independently of auxin. Furthermore, we demonstrate V-ATPase inhibition by SA application and a rapid increase of vacuole pH, potentially impairing basic cellular functions.

## Results

### SA specifically inhibits root growth

**Figure 1.**
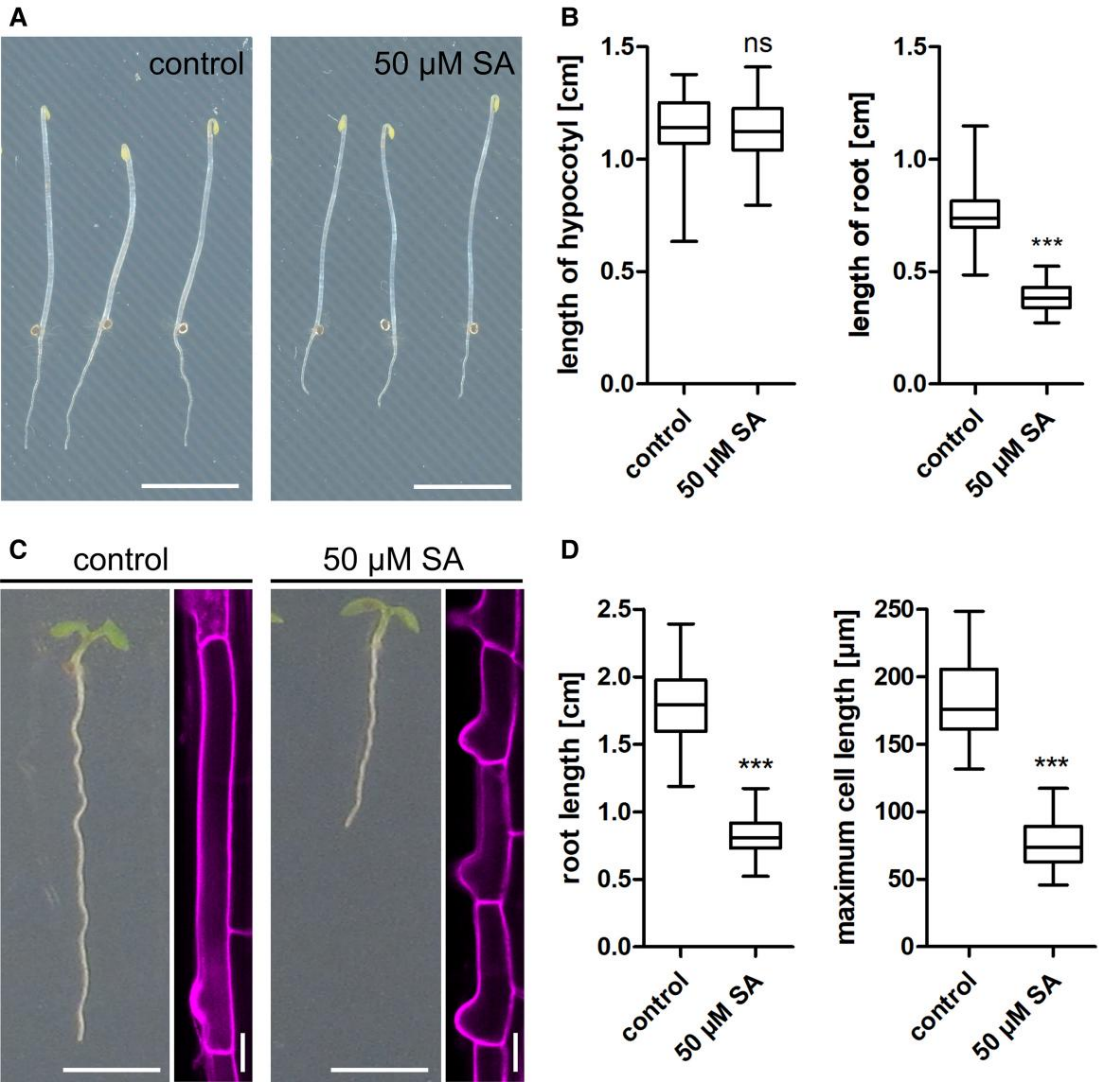
SA reduces root growth and cell size. **A)** Col-0 Arabidopsis seeds were placed on agar plates and kept in light for 8 h, then transferred to the dark for an additional 5 days. Scale bars: 0.5 cm. **B)** Length of root and hypocotyl after 5 days in the dark (hypocotyl: *n* control = 42, 50 *µ*M SA = 44; root: control = 42, 50 *µ*M SA = 44). Data is presented in a whisker plot with Student's *t*-test. ***P ≤ 0.001. Box limits represent 25th–75th percentile, the horizontal line the median and the whiskers minimum to maximum values. **C)** Representative images of nonetiolated Arabidopsis seedlings comparing root length and cell size upon SA treatment. Horizontal scale bars: 0.5 cm; vertical scale bars: 20 *µ*m. **D)** Quantification of root length (*n* control = 142, 50 *µ*M SA = 138) and maximum cell elongation (*n* control = 28, 50 *µ*M SA = 44) in 7-day-old Arabidopsis seedlings. Roots were stained with PI to highlight cell walls, and fully elongated cells were measured. Data are presented in a whisker plot with Student's *t*-test. ***P ≤ 0.001. Box limits represent 25th–75th percentile, the horizontal line the median and the whiskers minimum to maximum values.

Pathogen infection leads to the accumulation of several phytohormones. Upon infection with the necrotrophic fungus *Botrytis*, the defence-related hormones SA, jasmonic acid (JA) but also auxin are significantly upregulated (Supplementary Fig. S1, A to E). Recently, it has been demonstrated that SA impacts plant growth and development independently of its receptor NPR1 (Tan et al. 2020). By exogenously applying different SA concentrations on 7-day-old Arabidopsis seedlings, we observed a dosage-dependent root growth reduction (Supplementary Fig. S2, A and B). Treatment with 50 *µ*M SA inhibited root growth of the WT and the *npr1 npr3 npr4* triple mutant to the same extent (Zhang et al. 2006), by 50% (Supplementary Fig. S2C). Degradation of endogenous SA in the transgenic *nahG* line, expressing a bacterial salicylate hydroxylase gene (Friedrich et al. 1995), led to significantly less root growth inhibition (Supplementary Fig. S2D). Notably, this growth-inhibiting effect was only found in underground but not aerial tissue: Etiolated seedlings treated with 50 *µ*M SA displayed a 50% reduction in root length but no reduction of hypocotyl size (Fig. 1, A and B). To confirm responsiveness of the hypocotyl to pharmacological treatments, we used Concanamycin A (ConcA) as positive control. ConcA is known to prevent hypocotyl growth by specifically inhibiting vacuolar H^+^-ATPase activity. Indeed, application of ConcA showed a significant decrease of hypocotyl length at 0.2 *µ*M concentration (Supplementary Fig. S2E). Notably, the SA-induced root growth reduction correlated well with restriction of maximal size of root epidermal cells in the differentiation zone (Fig. 1, C and D).

### SA reduces cell size and impacts on auxin by manipulating PIN2

**Figure 2.**
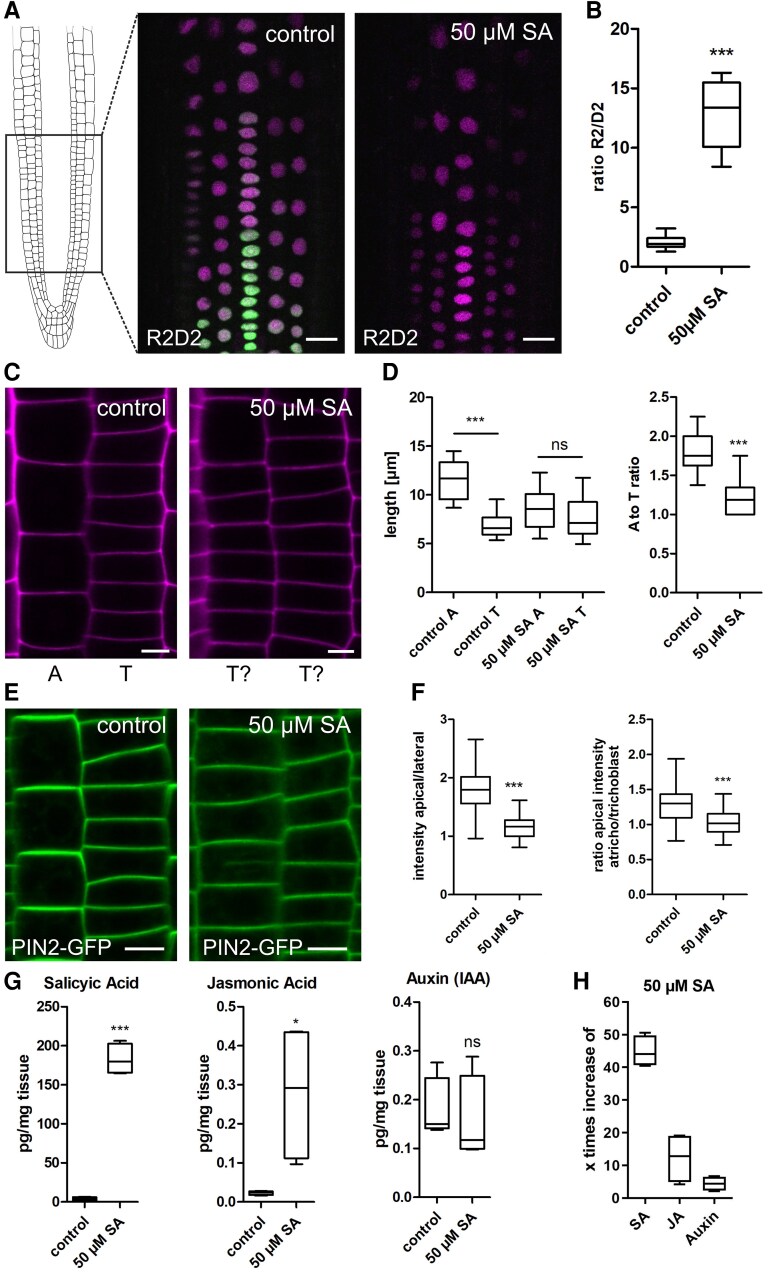
SA reduces cell size and alters PIN2 distribution. **A)** Area of root for microscopic experiments. Representative images of the rapid auxin-input reporter R2D2 before and after SA treatment. Scale bar: 30 *µ*m. **B)** Ratio calculation by dividing R2 by D2. Six-day-old R2D2 seedlings were transferred to ½MS + medium supplemented with 50 *µ*M SA and grown for an additional 24 h (*n* control = 17, *n* 50 *µ*M SA = 20). Data is presented in a whisker plot with Student's *t*-test. ***P ≤ 0.001. **C)** PI-stained epidermal cell files in the root meristem. A, Atrichoblast cell; T, Trichoblast cell. Scale bar: 5 *µ*m. **D)** Quantification of atrichoblast and trichoblast cell length changes after SA treatment. Six-day-old seedlings were transferred to ½MS + medium supplemented with 50 *µ*M SA and grown for an additional 24 h. The lengths of atrichoblast cells near the transition zone were measured and compared with neighboring trichoblast cells (length: *n* control A = 27, *n* control T = 27, *n* 50 *µ*M SA A = 22, *n* 50 *µ*M SA T = 22; ratio: *n* control = 27, *n* 50 *µ*M SA = 28). Data is presented in a whisker plot with Student's *t*-test. ***P ≤ 0.001. **E)** Trichoblast and atrichoblast cell files of the PIN2-GFP marker line before and after SA treatment. Scale bar: 9.5 *µ*m. **F)** Quantification of the PIN2-GFP intensity ratio of the apical and lateral side of atrichoblast cells. In further quantification, this ratio was divided by the ratio of the apical and lateral side of trichoblast cells. PIN2-GFP seedlings were grown on control plates for 4 days and transferred for 24 h on medium supplemented with 50 *µ*M SA (intensity: *n* control = 73, *n* 50 *µ*M SA = 66; ratio: *n* control = 36, *n* 50 *µ*M SA = 36). Data is presented in a whisker plot with Student's *t*-test. ***P ≤ 0.001. **G, H)** Six-day-old Col-0 seedlings were transferred to ½MS + medium supplemented with 50 *µ*M SA for an additional 24 h. Whole seedlings (60 mg) were used to measure SA, JA, and auxin (IAA) concentrations (all: *n* control = 4, *n* 50 *µ*M SA = 4). Data is presented in a whisker plot with Student's *t*-test. *P ≤ 0.05, ***P ≤ 0.001. Box limits in all whisker plots represent 25th–75th percentile, the horizontal line the median and the whiskers minimum to maximum values.

Reduced root cell size upon auxin treatment has been previously described (Löfke et al. 2015a). Auxin also affects root and shoot growth differentially, in a concentration dependent manner (Sauer et al. 2013). Consequently, we investigated a potential crosstalk between SA and auxin in epidermal cells of the root meristem. Here, the epidermis is regularly spaced into shorter tricho- and longer atrichoblast cells and represents a suitable model to study cell size differences (Berger et al. 1998; Löfke et al. 2013). To analyze auxin accumulation upon SA application, we used the semi-quantitative auxin-input reporter R2D2 (Liao et al. 2015). The reporter showed a significant reduction of ratio after 24 h SA treatment (Fig. 2, A and B), indicating elevated auxin levels. At the same time, we noticed that, in the time frame of 24 h, cell size differences of tricho -and atrichoblasts were diminished (Fig. 2C). In parallel, we observed an SA-induced increase of cell number which was accompanied by an increase in meristem size (Supplementary Fig. S3, A to C). To determine if this was the result of higher mitotic activity, the B1-type cyclin cell cycle marker *CYCB1;1::GUS* was used (Colón-Carmona et al. 1999). Indeed, upon 24 h SA treatment the reporter showed increased activity (Supplementary Fig. S3D), implying that a higher cell division rate might be responsible for the loss of cell size differences between atrichoblasts and trichoblasts.

**Figure 3.**
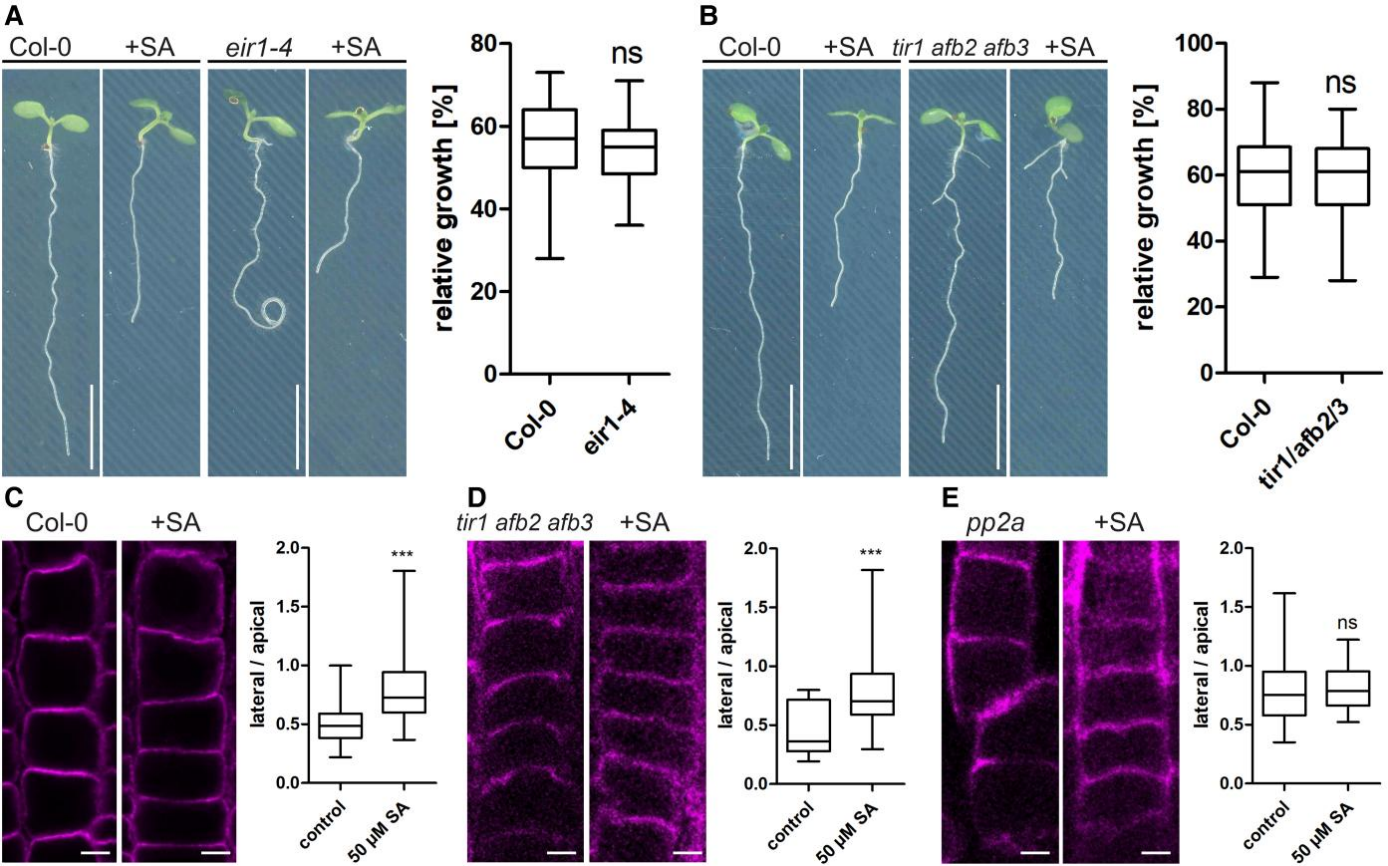
Neither the PIN2 mutant eir1-4 nor the auxin receptor mutant tir1 afb2 afb3 show increased resistance to SA. **A, B)** Col-0, *eir1-4*, and *tir1 afb2 afb3* seedlings were grown for 7 days on ½MS + plates supplemented with 50 *µ*M SA. Calculation of relative growth: root length of seedlings grown on SA is divided by mean root length of seedlings from control plates (*n* Col-0 = 21, *n* eir1-4 = 29; *n* Col-0 = 57, *tir1 afb2 afb3* = 59). Scale bars in all untreated lines also apply to the SA treatment: 40 mm. Data is presented in a whisker plot with Student's *t*-test. **C–E)** Immunolocalization of PIN2 in Col-0 (*n* control = 53, *n* 50 *µ*M SA = 82), tir1 afb2 afb3 (*n* control = 16, *n* 50 *µ*M SA = 152), and pp2a (*n* control = 20, *n* 50 *µ*M SA = 36). Quantification of the intensity ratio of the apical and lateral side of atrichoblast cells. Scale bar: 4 *µ*m. Data is presented in a whisker plot with Student's *t*-test. ***P ≤ 0.001. Box limits in all whisker plots represent 25th–75th percentile, the horizontal line the median and the whiskers minimum to maximum values.

It has been shown that both cell types exhibit different accumulation of the auxin efflux carrier PIN2 (Löfke et al. 2015b). It was suggested that higher PIN2 concentration observed in atrichoblasts could result in increased auxin export rates and, hence, less cell size restriction. Likewise, lower PIN2 abundance in trichoblasts could slow down auxin export, increasing its inhibitory effect. To test for differences in the distribution of PIN2 in tricho-and atrichoblast cells upon SA treatment we used a functional PIN2-GFP fusion under the endogenous promoter (Abas et al. 2006). Under control conditions, the fluorescence intensity of PIN2-GFP at the apical PM in atrichoblasts was higher than that in trichoblast cells as shown before (Löfke et al. 2015b). SA application, however, abolished this difference, resulting in a more uniform PIN2 distribution at the PM of atrichoblasts (Fig. 2, E and F). This led to the assumption that auxin is responsible for the reduction in cell size following SA treatment. Since auxin-induced cell size restrictions might be regulated by the size of the plant vacuole (Löfke et al. 2015a; Scheuring et al. 2016), we speculated that SA-induced inhibition of cell size functions similarly. However, as we measured phytohormone levels upon exogenous application of 50 *µ*M SA in Arabidopsis seedlings, we found a co-accumulation of SA with JA, but not with auxin (Fig. 2, G and H). To test whether the PIN2-dependent changes of auxin localization or canonical auxin perception is causative, we used the PIN2 mutant *eir1-4* and the auxin receptor triple mutant *tir1 afb2 afb3* and tested SA-induced root growth inhibition. Both mutants were sensitive to SA treatment and accordingly root length inhibition did not differ from that of Col-0 control plants (Fig. 3, A and B).

Using a PIN2 antibody, we also determined PIN2 polarity upon SA application in Col-0 wt and the *tir1 afb2 afb3* mutant. Immunolocalization displayed a more uniform PIN2 distribution in SA-treated roots similar to the findings with the PIN2-GFP line (Fig. 3, C and D). Since SA has been shown to directly bind to PP2A (Tan et al. 2020), we also tested root length and PIN2 polarity in a *pp2a* knockout line: Compared with wildtype, root growth was more severely affected after SA application and PIN2 polarity was diminished in untreated conditions (Fig. 3E), confirming the reported SA hypersensitivity of *pp2a* (Tan et al. 2020).

### SA changes vacuolar morphology and reduces SNARE abundance

**Figure 4.**
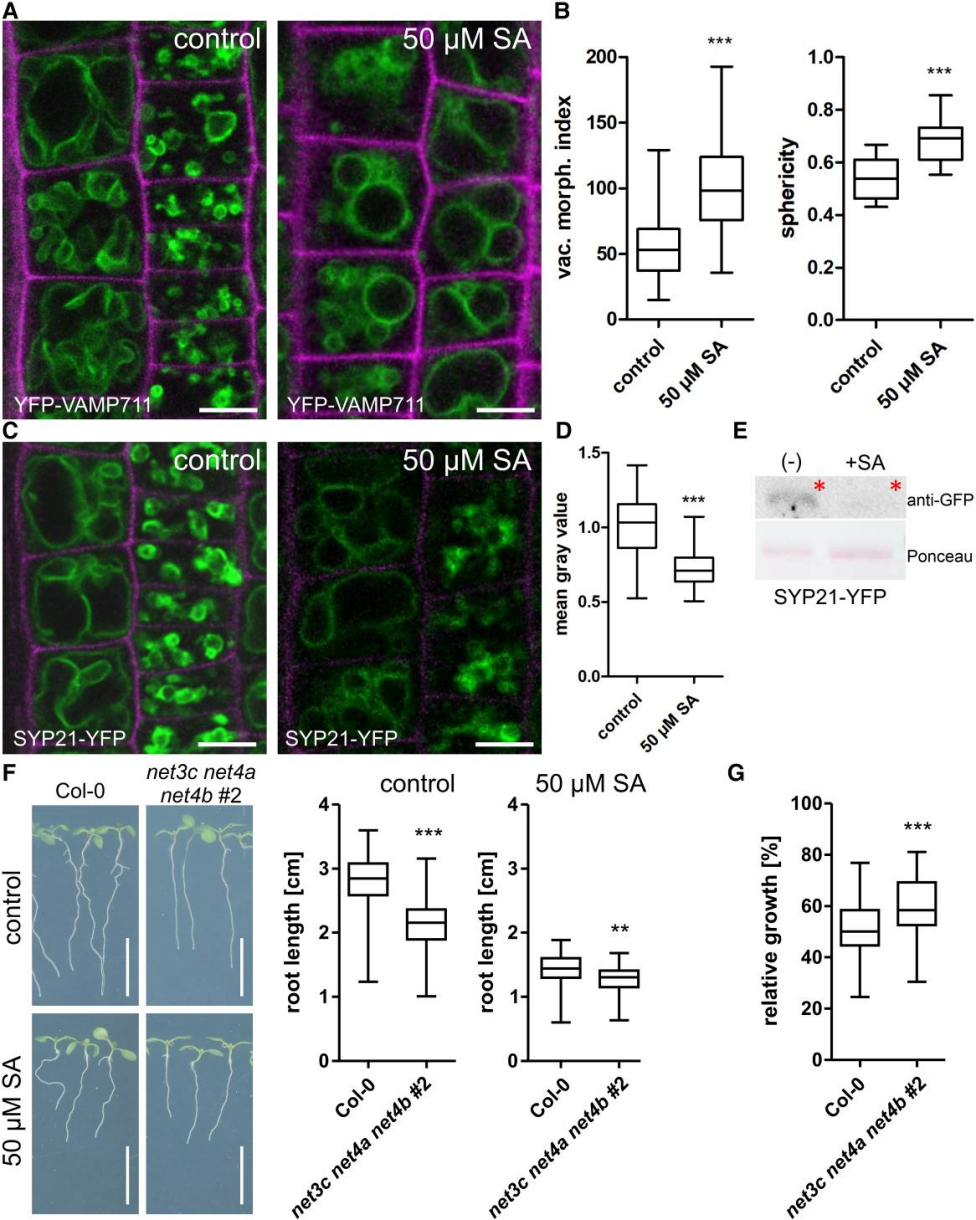
SA rapidly changes vacuolar morphology. **A)** The tonoplast marker line YFP-VAMP711 was used to visualize vacuole changes after 24 h SA treatment (50 *µ*M). Scale bar: 14 *µ*m. **B)** VMI was calculated as a proxy for vacuole size (*n* control = 64, *n* 50 *µ*M SA = 70). Sphericity was quantified using the Imaris software (Oxford Instruments) on 3D vacuole models (*n* control = 14, *n* 50 *µ*M SA = 15) shown in Fig. 5. Data is presented in a whisker plot with Student's *t*-test. ***P ≤ 0.001. **C)** Representative images of the SNARE marker line SYP21-YFP before and after 24 h SA treatment (50 *µ*M). Scale bar: 15 *µ*m. **D)** Quantification of SYP21-YFP intensity. SA-treated samples were normalized to DMSO control (*n* control = 42, *n* 50 *µ*M SA = 39). Data is presented in a whisker plot with Student's *t*-test. ***P ≤ 0.001. **E)** Western blot detection of SYP21-YFP levels in seedlings using GFP antibodies. Red asterisks indicate expected size on the membrane. **F)** Root length of 7-day-old seedlings grown on ½ MS + agar control plates (*n* Col-0 = 54, *n net3c net4a net4b #2* = 56) and plates supplemented with 50 *µ*M SA (*n* Col-0 = 56, *n net3c net4a net4b #2* = 51). Data is presented in a whisker plot with Student's *t*-test. ***P ≤ 0.001, **P ≤ 0.01. Scale bar: 1 cm. **G)** Calculation of relative growth: root length of seedlings grown on SA is divided by mean root length of seedlings from control plates (*n* Col-0 = 56, *n net3c net4a net4b #2* = 51). Values are given as percentages. Data is presented in a whisker plot with Student's *t*-test. ***P ≤ 0.001. Box limits in all whisker plots represent 25th–75th percentile, the horizontal line the median and the whiskers minimum to maximum values.

To further investigate the reason for SA-induced root cell growth inhibition, we investigated the effect of SA treatment on vacuolar morphology. Using the *pUBQ10::YFP-VAMP711* as marker to visualize the tonoplast, we observed a change of vacuolar morphology within minutes after SA treatment: vacuoles in epidermal cells of the root meristem displayed homotypic fusion events and became almost entirely spherical within 4 h (Supplementary Videos S1 and S2). To quantify these changes, we used the vacuolar morphology index (VMI) to estimate vacuole size (Löfke et al. 2015a). SA application increased the VMI significantly (Fig. 4, A and B), while treatments with the SA analogs Acibenzolar-S-methyl (BTH) and 2,6-Dichloroisonicotinic acid (INA) had no effect (Supplementary Fig. S4A and B). Additionally, treatment of seedlings with 50 *µ*M INA and BTH, having comparable pKa values, displayed no root growth inhibition (Supplementary Fig. S4C and D). Notably, SA treatment resulted in a different vacuolar phenotype compared with auxin-induced changes. While SA-induced more fused and hence, round vacuoles, auxin treatment led to more infolded, constricted vacuoles with a decreased VMI (Löfke et al. 2015a; Scheuring et al. 2016). As the abundance of soluble NSF Attachment Protein Receptor (SNARE) complexes at the vacuole have been shown to mediate the observed auxin effect, we tested whether SNAREs are also involved in SA-induced changes of vacuolar morphology. To this end, we examined the abundance of the SNARE SYP21-YFP upon SA treatment using confocal microscopy and immunodetection. Exogenous application of 50 *µ*M SA decreased SYP21-YFP fluorescence intensity in roots and reduced SYP21-YFP protein levels in whole seedlings (Fig. 4, C to E). To strengthen this, we tested a mutants with impaired vacuole fusion machinery, the *net3c net4a net4b* triple mutant. (Kaiser et al. 2023). When treated with SA, root length of *net3c net4a net4b* was less inhibited than the corresponding Col-0 control (Fig. 4F). This indicates a partial resistance against SA treatment (Fig. 4G) and connects the vacuole phenotype to growth inhibition.

### Short-term SA application does not impact vacuolar occupancy

**Figure 5.**
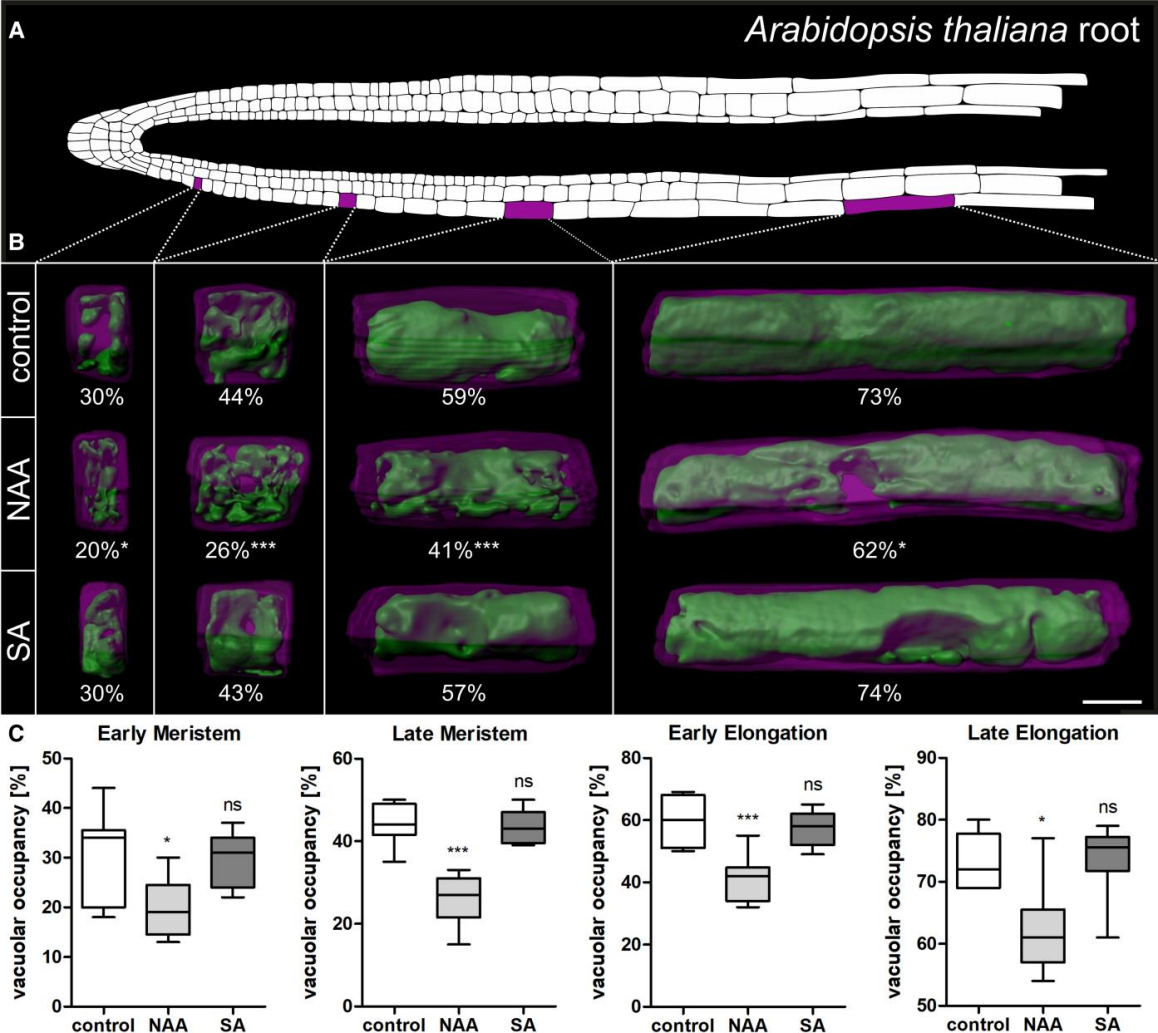
Short-term SA application does not impact vacuolar occupancy. **A)** Schematic representation of an *Arabidopsis thaliana* root showing epidermis, cortex, and endodermis cell files. The investigated regions are marked in magenta. **B)** Six-day-old seedlings were grown on ½MS + medium and transferred for 24 h to medium supplemented with 50 *µ*M SA and 250 nm NAA. The seedlings were stained with PI and BCECF, and *z*-stacks were acquired. Using these, 3D reconstructions of the vacuole (green) and its corresponding cell (magenta) were generated, and relative vacuole size (vacuolar occupancy) was quantified. The different columns represent (from left to right): early meristem, late meristem, early elongation, and late elongation zones. The numbers under each picture indicate the mean percentage of vacuolar occupancy. For comparison, student's *t*-tests were carried out (control = vacuoles from untreated seedlings): *P ≤ 0.05, ***P ≤ 0.001. Scale bar: 10 *µ*m. The recess seen in many models indicates the position of the nucleus. **C)** Quantification of vacuolar occupancy in the 4 root regions: early meristem (*n* control = 9, *n* NAA = 10, *n* SA = 8), late meristem (*n* control = 9, *n* NAA = 9, *n* SA = 9), early elongation (*n* control = 9, *n* NAA = 7, *n* SA = 8), and late elongation zones (*n* control = 8, *n* NAA = 10, *n* SA = 8). Data is presented in a whisker plot with Student's *t*-test. *P ≤ 0.05, ***P ≤ 0.001. Box limits represent 25th–75th percentile, the horizontal line the median and the whiskers minimum to maximum values.

Despite the distinct changes of vacuolar morphology after SA treatment, it was still unclear if SA affects vacuolar size. To determine this, we used the luminal vacuole dye BCECF in combination with propidium iodide (PI), staining the cell wall, for 3D imaging (Scheuring et al. 2015). To this end, we captured *z*-stacks of root cells from 4 different positions, including the early -and late meristem, as well as the early and -late elongation zone (Fig. 5A). Relative vacuole size (vacuolar occupancy) was determined by calculating vacuolar volume and corresponding cellular volume based on the respective 3D models. Since it has been shown before that auxin decreased vacuolar occupancy in meristematic cells (Scheuring et al. 2016), it was included as positive control. Remarkably, auxin treatment decreased vacuolar occupancy in cells from all positions, while SA treatment did not change the relative vacuole size at any position (Fig. 5, B and C). However, SA- induced changes of vacuolar morphology were still evident in the 3D models (Fig. 5B), indicating that morphology but not the relative size of the vacuole is affected. Notably, when seedlings were grown on SA for 7 days we indeed could observe a reduction of vacuolar occupancy in the early -and late meristem, as well as the early elongation zone (Supplementary Fig. S5, A to C). Decrease of vacuolar occupancy, however, was not as strong as the auxin-induced decrease at all positions and do not explain the cellular and molecular phenotypes observed after short-term SA application (Figs. 1–4).

The general SA-induced changes of the vacuole to a more spherical shape was observed in all experiments and was somewhat reminiscent of the phenotype resulting from the gene knockout of 3 tonoplast proton pumps: *fugu5-1 vha-a2 vha-a3* (Kriegel et al. 2015). The triple mutant is lacking V-ATPase and V-PPase activity resulting in a significantly higher vacuolar pH which is also already observed for the *vha-a2 vha-a3* double mutant.

**Figure 6.**
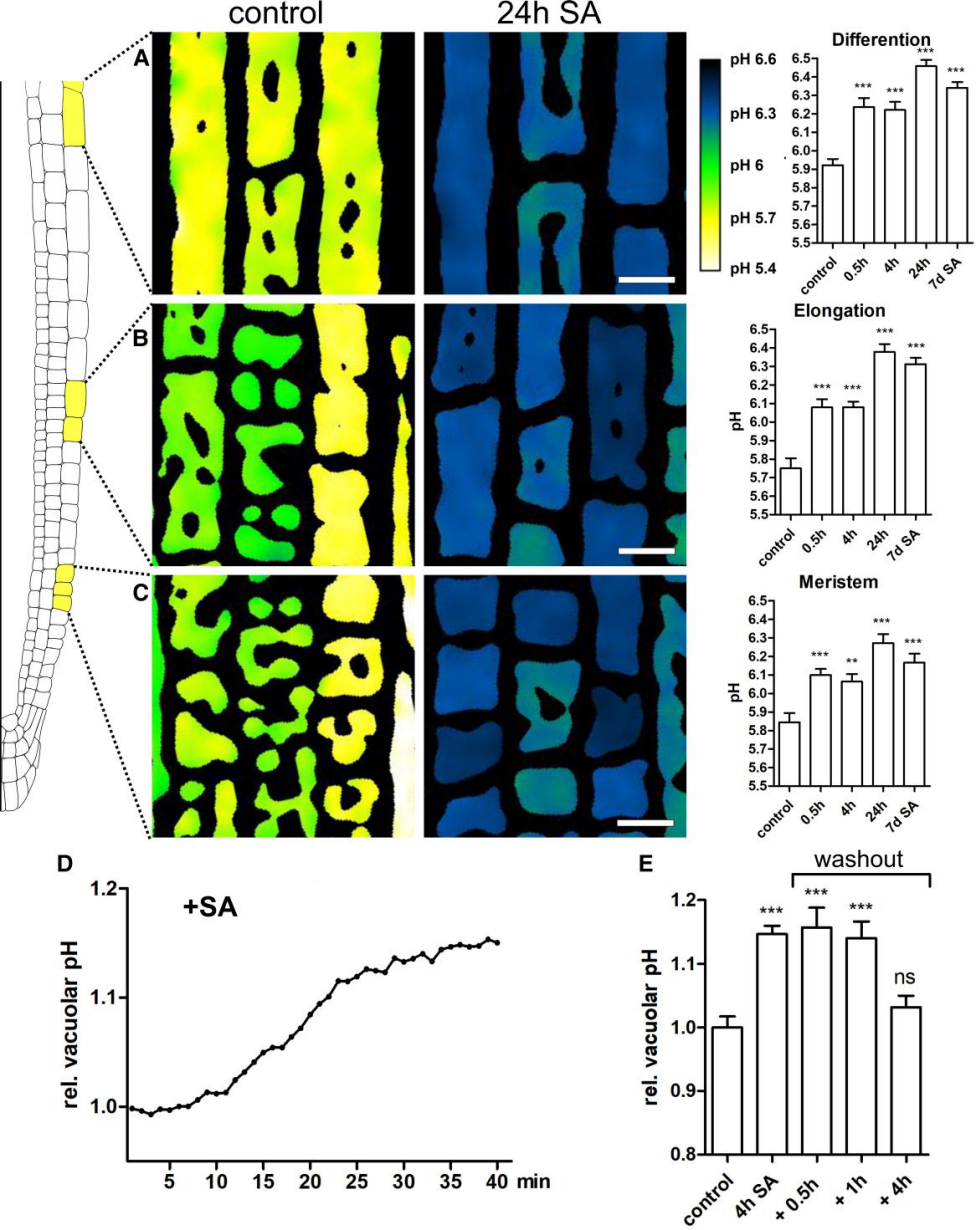
SA rapidly increases vacuolar pH. **A–C)** Col-0 seedlings were grown on ½MS + plates and treated with 50 *µ*M SA for 0.5 h, 4, 24 h, and 7 days. Seedlings were stained with BCECF, and ratiometric images were created by dividing the λex 488 intensity by the λex 458 intensity. Emission ratios were converted to pH values by employing an in situ calibration. Images were captured in 3 different root regions: the late meristem (*n* control = 23, *n* 0.5 h = 18, *n* 4 h = 20, *n* 24 h = 23, *n* 7d SA = 21), the early elongation zone (*n* control = 22, *n* 0.5 h = 22, *n* 4 h = 22, *n* 24 h = 25, *n* 7d SA = 22), and the differentiation zone (*n* control = 21, *n* 0.5 h = 20, *n* 4 h = 23, *n* 24 h = 25, *n* 7d SA = 22). Scale bar: 20 *µ*m. The scale bar shown in “24 h SA” always applies to the control picture as well. Data was analyzed using ANOVA with Tukey post-hoc test. **P ≤ 0.01, ***P ≤ 0.001. Means are shown with standard error. **D)** 6-day-old seedlings were grown on ½MS + medium, stained with BCECF and transferred to medium containing 50 *µ*M SA. Shown is one representative recording displaying relative vacuolar pH by normalizing the λex 488/λex 458 ratio to the timepoint of 0 min. **E)** Col-0 seedlings were grown on ½MS + plates for 6 days and treated with 50 *µ*M SA for 4 h. For washout experiments seedlings were transferred to liquid ½ MS + media for 0.5, 1 and 4 h. Relative vacuolar pH was calculated by normalizing the λex 488/λex 458 ratio to the DMSO control (*n* control = 21, *n* 4 h SA = 27, *n* + 0.5 h = 13, *n* + 1 *h* = 26, *n* 4 h = 10). Data was analyzed using ANOVA with Tukey post-hoc test. ***P ≤ 0.001, ns = not significant. Means are shown with standard error.

To assess vacuolar pH directly, we used the previously employed vacuole dye BCECF and carried out ratiometric measurements upon exogenous SA application. Emission ratios were converted to pH values by employing an in situ calibration using 6–8 stained roots for every test point on the curve (Supplementary Fig. S6A). Already after 30 min of SA treatment, a significant increase in vacuolar pH was detected (Supplementary Fig. S6B). This effect lasted for all tested time points, including seedling grown on SA containing media for 7d. For detailed analysis, we determined SA-induced pH changes in the late meristem, the elongation and the differentiation zone (Fig. 6, A to C). Changes were statistically significant in all root regions and the highest increase was from pH 5.75 to 6.39 observed in the elongation zone. To understand the temporal dynamics of the SA-induced pH increase, we measured pH directly upon exposure to 50 *µ*M SA. After 10–15 min we observed a steep pH increase which reached a plateau at around 30 min (Fig. 6D). To address reversibilty, we measured vacuolar pH in seedlings after 4 h SA treatment and subsequently transferred them into fresh medium without SA, measuring pH again after 30 min, 1 and 4 h. The significant increase of vacuolar pH after SA application was fully reverted 4 h upon transfer to fresh medium (Fig. 6E).

We hypothesized that one possible explanation for the SA-induced pH increase could be the inactivation of SA by transport into the vacuole. In tobacco suspension cells it has been observed that glycosylated SA is transported into the vacuole, probably by using a H^+^-antiport mechanism (Dean et al. 2005). In case such a transport mechanism would exist in Arabidopsis, we expected application of SA to increase levels of glycosylated SA in the vacuole at the expense of H^+^. To follow this hypothesis, we tested a knockout and an overexpression line of the glucosyltransferases UGT76B1. It has been shown that loss of UGT76B1 mimics high SA levels while overexpression of UGT76B1 depletes levels of free SA (Bauer et al. 2021). Root growth assays revealed that the overexpression line was indeed more resistant than the Col-0 control, whereas *ugt76B1* was significantly more sensitive to SA (Supplementary Fig. S7, A and B). However, since vacuolar pH was increased similarly to the Col-0 control in both lines (Supplementary Fig. S7C), glycosylation certainly impacts SA response but pH increase and change of vacuolar morphology seems to occur independently.

**Figure 7.**
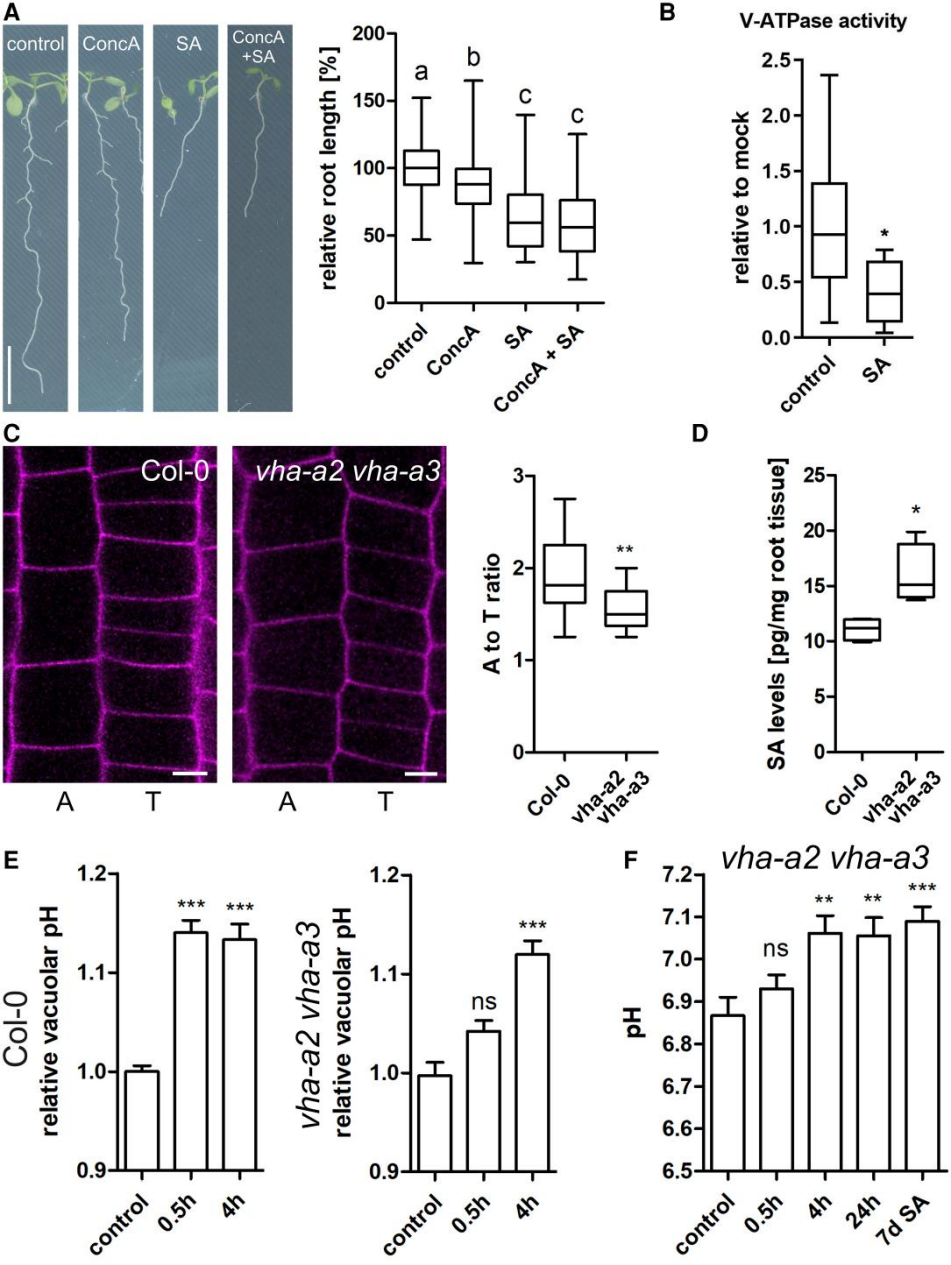
SA inhibits V-ATPase activity. **A)** Root growth was tested in response to the V-ATPase inhibitor Concanamycin A (ConcA; 75 nm), SA (50 *µ*M) or a combination of both (*n* control = 148, *n* ConcA = 153, *n* SA = 147, *n* ConcA + SA = 151). Scale bar: 0.7 cm. Statistical analysis was performed using a one-way ANOVA test with Tukey post-hoc test. Change in letter equals *P* ≤ 0.001. **B)** Four-day-old seedlings were grown on ½MS + medium and then transferred to liquid ½MS + medium for 2 wk. The seedlings were treated with 50 *µ*M SA for 5 h, and microsomal fractions were collected from total protein extracts by ultracentrifugation (*n* control = 9, *n* SA = 9). The ATP hydrolytic activity of the NaNO₃-sensitive V-ATPase was measured by quantifying phosphate-malachite complexes as a proxy for the released inorganic phosphate. Data is presented in a whisker plot with Student's *t*-test. *P ≤ 0.05. **C)** PI-stained epidermal cell files in root meristem of 7-day-old seedlings. A = Atrichoblast cell; T = Trichoblast cell. Scale bar: 5 *µ*m. The lengths of atrichoblast cells near the transition zone were measured and compared with adjacent trichoblast cells by doing a ratio quantification (*n* Col = 20, *n vha-a2 vha-a3* = 15). Data is presented in a whisker plot with Student's *t*-test. **P ≤ 0.01. **D)** SA concentration was measured in Col-0 and *vha-a2 vha-a3* (*n* Col-0 = 4, *n* SA = 4). Data is presented in a whisker plot with Student's *t*-test. *P ≤ 0.05. Box limits in the whisker plots (A–D) represent 25th–75th percentile, the horizontal line the median and the whiskers minimum to maximum values. **E)** Col-0 and *vha-a2 vha-a3* seedlings were grown on ½MS + plates for 6 days and treated with 50 *µ*M SA for 0.5 and 4 h Quantification of relative vacuolar pH was performed using BCECF by normalizing the λex 488/λex 458 ratio to the DMSO Col-0 control (*n* control = 44, *n* 0.5 *h* = 25, *n* 4 h = 18) and the a2a3 control (*n* control = 25, *n* 0.5 h = 14, *n* 4 h = 22). Data was analyzed using ANOVA with Tukey post-hoc test. ***P ≤ 0.001. Means are shown with standard error. **F)**  *vha-a2 vha-a3* seedlings were grown on ½MS + plates for 6 days and treated with 50 *µ*M SA for 0.5 h, 4 h, 24 h or grown 7 days on it. Seedlings were stained with BCECF and the λex 488 intensity divided by the λex 458 intensity was measured. Absolut pH values were calculated based on a calibration curve (*n* control = 18, *n* 0.5 h = 17, *n* 4 h = 16, *n* 24 h = 17, *n* 7d SA = 16). Data was analyzed using ANOVA with Tukey post-hoc test. **P ≤ 0.01, ***P ≤ 0.001. Means are shown with standard error.

Next, we tested whether V-ATPase function is involved in SA-induced pH increase. To this end, we pharmocologically inhibited rooth growth by application of the V-ATPase inhibitor ConcA and SA. Both drugs inhibited root growth significantly but combining them did not further increase the effect (Fig. 7A). In the next step, we directly tested V-ATPase activity by preparing microsomal fractions from Arabidopsis seedlings and measured phosphate release as proxy. Indeed, 5 h 50 *µ*M SA treatment significantly inhibited V-ATPase activity (Fig. 7B), providing an explanation for the observed pH increase.To strengthen this link, we assessed cell size differences of atrichoblasts and trichoblasts in a partial V-ATPase mutant. Due to poor growth of the *fugu5-1 vha-a2 vha-a3* triple mutant (Kriegel et al. 2015), we opted for the V-ATPase double mutant *vha-a3 vha-a3* (Krebs et al. 2010). Notably, not only atrichoblast-trichoblast differences were smaller in the double mutant (Fig. 7C) but also SA levels were significantly elevated (Fig. 7D). Measuring vacuolar pH in *vha-a2 vha-a3* showed a slightly delayed increase (Fig. 7E) with a significantly lower amplitude. While the pH in the Col-0 control increased by 0.5–0.6 values, the pH of *vha-a2 vha-a3* only increased by 0.3 (Fig. 7F). Together, this indicates a partial resistance of *vha-a2 vha-a3*, strengthening the finding that SA inhibits V-ATPase activity.

## Discussion

Only recently, the function of the phytohormone SA within plant growth and development came into focus. Previously, many studies described a growth-inhibiting effect by high exogenous or endogenous SA levels (Rivas-San Vicente and Plasencia 2011; Pokotylo et al. 2022). In some cases, this inhibition was explained by diminished resources due to the classical *growth-defence trade-off*: the plant's energy pool needs to be used for all arising tasks. In extreme situations, e.g. pathogen attack, energy resources must be invested for defence reactions but, ergo, are not sufficient anymore to maintain optimal growth (Huot et al. 2014). More recently, it was proposed that low SA concentrations (<50 *µ*M) in Arabidopsis might have a role in growth and development, and only higher concentrations contribute to plant defence (Pasternak et al. 2019). However, in Arabidopsis and tobacco it has been shown that relatively low concentrations of SA and its functional analogues INA and BTH are sufficient to induce SAR (Ward et al. 1991; Delaney et al. 1994; Lawton et al. 1996) and thus it seems plausible that growth-inhibition and activated plant defence occur simultaneously. While they may occur simultaneously, the effect of SA on growth and immunity seems to involve distinct signaling pathways. Indeed, signaling of the SA receptors NPR1, 3 and 4 is not required for the transmission of SA-mediated root growth inhibition (Supplementary Fig. S2) (Tan et al. 2020). This suggests that another SA-triggered signaling pathway, potentially specifically regulating growth, might exist.

Another explanation might be the observed auxin-SA crosstalk: lateral root initiation, increased cell division rates and irregular cell file development all correspond to changes triggered by auxin (Pasternak et al. 2019). Interestingly, overall auxin levels do not increase upon SA treatment (Fig. 2), pointing towards another mechanism to regulate growth. It has been shown that SA directly binds to a phosphatase (PP2a) which in turn regulates the polarity and thereby activity of the auxin efflux carrier PIN2 (Tan et al. 2020). Additionally, also other cellular functions, such as endocytosis, are affected by SA (Du et al. 2013). This raises the question to which extent specific SA functions can be mediated by auxin crosstalk alone. Using the R2D2 auxin reporter, we indeed detected strong auxin accumulation in response to SA (24 h) treatment in meristematic root cells (Fig. 2). Moreover, inhibition of cell elongation and loss of cell size differences in tricho-and atrichoblasts upon SA treatment resemble effects induced by exogenous auxin application (Löfke et al. 2013, 2015a). However, quantification of phytohormone levels in seedlings did not show a general accumulation of auxin after SA treatment (Fig. 2) although we cannot exclude that these changes are only cell-type specific. On the other hand, the findings that the auxin receptor triple mutant *tir1 afb2 afb3* and the PIN2 mutant *eir1-4* are fully sensitive to SA-induced root inhibition (Fig. 3), argue against auxin being solely responsible to conduct SA impact. The similarity of effects (e.g. inhibition of cell elongation) might be due to SA binding of auxin signaling components like PP2A (Tan et al. 2020). However, SA seems to act at least partially independent from nuclear auxin signaling. In line with this, we observe SA-induced changes of vacuolar morphology which differs significantly from auxin-induced changes: while SA seems to induce homotypic vacuole fusion, auxin treatment led to highly constricted vacuoles with decreased relative size (Löfke et al. 2015a; Scheuring et al. 2016). The contrasting effect of both phytohormones on the abundance of the SNARE SYP21 support this as well. The partial resistance of the *net3c net4a net4b* triple mutant, which possesses fragmented vacuoles, hints towards a correlation between homotypic vacuole fusion and root growth inhibition. On the other hand, the relative size (vacuolar occupancy) of SA-treated vacuoles did not decrease questioning the space-filling function of the vacuole to be decisive to regulate growth here (Scheuring et al. 2016; Krüger and Schumacher 2018; Kaiser and Scheuring 2020; Kaiser et al. 2021). However, the observed pH increase occurred in parallel to homotypic vacuole fusions within minutes. Initially, we suspected increased vacuolar pH to be related to the activity of H^+^-antiporters. In tobacco cells, transport of glycosylated SA into the vacuole occurs via an H^+^-antiport mechanism which is energized by the proton gradient between the vacuole and the cytosol (Dean et al. 2005). In Arabidopsis, glycosylated SA has been detected in vacuolar membrane-enriched vesicles and stimulated by MgATP, whereas vanadate (an ABC transporter inhibitor) and bafilomycin A_1_ (a vacuolar H^+^-ATPase inhibitor) inhibited uptake (Vaca et al. 2017). This suggests that glycosylated SA involves both an ABC transporter and an H^+^-antiporter. In line with this, knockout and overexpression of the small-molecule glucosyltransferases UGT76B1 (von Saint Paul et al. 2011) resulted in differential sensitivity in respect to SA-induced root growth inhibition (Supplementary Fig. S7). However, vacuolar pH in these lines reacts similarly to the Col-0 control in response to SA application Supplementary Fig. S7. This indicates that glycosylation of SA represents an efficient mechanism to deactivate the phytohormone but it fails to explain the observed alkalization of the vacuole.

Since V-ATPase activity is crucial for maintaining the acidic pH in the vacuole, we compared the effect of the V-ATPase inhibitor ConcA with SA. To this end, we measured root length upon ConcA or SA treatment and when both were combined. Individually, ConcA and SA both inhibited root growth significantly but their combination did not result in an additive effect (Fig. 7A). This could indicate that ConcA and SA work by using the same mode of action. Consequently, this would suggest that SA impacts directly V-ATPase activity. However, we cannot rule out that 1 drug interferes with the activity of the second drug. To follow the assumption that SA inhibits V-ATPase activity directly, we measured V-ATPase activity and found a significant decrease after SA treatment. Delving deeper, we could show that atrichoblast-trichoblast differences in *vha-a2 vha-a3* are smaller than in the Col-0 control. This phenocopies the effect of SA on cell size and encouraged us to measure the SA concentration in the double mutant. Notably, SA levels were significantly elevated which is in line with the diminished atrichoblast and trichoblast cell size differences. Moreover, the SA effect on vacuolar pH was significantly reduced in *vha-a2 vha-a3* (Fig. 7). This suggests that a substantial part of the observed increase in vacuolar pH is due to inhibition of tonoplast-localised V-ATPase complexes containing VHA-a2 and VHA-a3 subunits.

Taken together, SA inhibits growth by limiting cell elongation. The size limitations are accompanied by changes of vacuolar morphology and a rapid increase in vacuolar pH, which is mediated by a reduction in V-ATPase activity. By interfering with V-ATPase function, SA might directly impact basic cellular functions such as nutrient storage in the vacuole and general vesicle trafficking (Krebs et al. 2010). The central role for V-ATPases in plant growth and development was established decades ago (Schumacher et al. 1999). SA-induced inhibition of V-ATPase activity, however, represents a different mechanism to regulate plant growth, potentially as part of the stress response.

## Materials and methods

### Plant material and growth conditions

*Arabidopsis thaliana* (Arabidopsis hereafter) ecotype Columbia-0 (Col-0) was used as wild type control. The following marker lines were described previously: R2D2 (Liao et al. 2015), PIN2-GFP (Abas et al. 2006) YFP-VAMP711 (Wave 9Y) (Geldner et al. 2009), SYP21-YFP (Robert et al. 2008). The following mutant and transgenic lines were described earlier: *nahG* (Friedrich et al. 1995), *npr1-1 npr3-1 npr4-3* triple mutant (*npr1 npr3 npr4*) (Zhang et al. 2006), *eir1-4* (Abas et al. 2006), *pp2aa1-6* (*pp2a*) (Blakeslee et al. 2008), *vha-a2 vha-a3* (Krebs et al. 2010), UGT76B1^OE^ and *ugt76b1b1* (von Saint Paul et al. 2011), *net3c net4a net4b* (Kaiser et al. 2023). Seeds were surface sterilized with ethanol and plates were stratified at 4 °C for 1–2 days in the dark and grown vertically at 22 °C under a 16 h light/8 h dark-cycle. For seedling growth, half-strength Murashige and Skoog (MS) medium (Duchefa, Netherlands), including 1% (w/v) sucrose (Roth, Germany), 2.5 mm MES (Duchefa) and 1% (w/v) Phytoagar (Duchefa) was used at pH 5.7.

### Cultivation of *Botrytis* and infection tests

*Botrytis cinerea* (*Botrytis* throughout the manuscript) was cultivated, harvested and use for infection tests as described previously (Jeblick et al. 2023).

### Chemicals and treatments

The dyes PI and BCECF-AM were acquired from Life Technologies (CA, USA). The synthetic auxin α-Naphthaleneacetic acid (NAA), SA and its analogues BTH and INA were obtained from Duchefa (Netherlands). Except PI, all chemicals were dissolved in dimethyl sulfoxide (DMSO).

### Confocal microscopy

Live cell imaging was performed using a Zeiss LSM880, AxioObserver SP7 confocal laser-scanning microscope, equipped with either a Zeiss C-Apochromat 40×/1.2 W AutoCorr M27 water-immersion objective or a Plan-Apochromat 20×/0.8 M27 objective (INST 248/254-1). Vacuole staining was carried out as described previously (Scheuring et al. 2015). Cell walls were stained by mounting roots in 0.01 mg/mL PI solution. Fluorescence signals were acquired for GFP/YFP/BCECF (excitation/emission 488 nm/500–571 nm), PI (excitation/emission 543 nm/580–718 nm), and processed using Zeiss software ZEN 2.3 or Fiji software (https://imagej.net/Fiji). Laser intensity was set to maximal 2% and the gains were adjusted in accordance with protein expression level and staining intensity. Z-stacks were recorded with a step size of 500 nm (for 3D reconstruction of cells and vacuoles to assess vacuolar occupancy).

### Phenotype analysis

For quantification of root length, Arabidopsis seeds were evenly placed on solid ½ MS + plates and afterwards subjected to stratification overnight at 4 °C before being transferred to the growth chamber. After a 7-day growth period, absolute root length was measured. For relative root length, the tested mutant or condition was normalized to the mean root length of the control. Calculation of VMI (vac. morph. index, VMI) and vacuolar occupancy of the cell were carried out as described previously (Kaiser et al. 2019). Meristem length was measured by staining 7-day-old seedlings with PI for 20 min, followed by image acquisition using a CLSM microscope. The distance between the quiescent center and the first elongating epidermal cell was quantified and determined as meristem length. For measuring hypocotyl length, seeds were sown on plates and exposed to light conditions for 8 h to initiate germination. Subsequently, the plates were kept in the dark for an additional 5 days at a constant temperature before documentation. Hypocotyl length was measured using ImageJ by determining the distance from the base of the seedling to the cotyledons. The length of atrichoblasts and trichoblasts was measured by taking CLSM pictures of the epidermal meristem. Here, the length of 3rd, 4th, 5th and 6th atrichoblasts, positioned before the first elongated cell (twice as high as wide), were measured and averaged. Subsequently, the number of the corresponding trichoblasts in the adjacent cell file was counted and averaged to the length of the atrichoblasts. The ratio between the averaged lengths of atrichoblasts and trichoblasts was then calculated for comparative analysis. Maximum cell length was conducted in elongated epidermal root hair cells (trichoblasts) on median confocal sections. To estimate the precise position for cell length measurements in the elongation zone, seedlings were stained with PI (0.02 mg/mL) for at least 20 min. Subsequently, images were acquired at positions where PI was not entering the vasculature anymore, indicating a differentiated endodermal diffusion barrier. PIN2 polarity was quantified using ImageJ by measuring the intensity of PIN2 fluorescence on the apical and lateral sides of the cells. Measurements were taken from 3 cells per root. The polarity ratio was calculated by dividing the intensity on the apical side by that on the lateral side. To assess differences between cell types, the polarity ratios of atrichoblasts and trichoblasts were further divided to determine the relative differences in PIN2 distribution between these 2 cell types.

### Quantification of phytohormones

Arabidopsis seedlings were grown for 6 days on ½MS + medium and an additional 24 h on ½MS + medium supplemented with 50 *µ*M SA or for 14 days on ½MS + medium and inoculated with spore suspension for 72 h. The whole seedlings were then flash frozen in liquid nitrogen and ground with a mortar and pestle until everything was a homogenous powder. 60 mg were weight and analyzed as described previously (Jeblick et al. 2023) at the HMLS Research Core Facilities in Heidelberg, Germany. For specific analysis of root hormone levels, the roots of 7-day-old seedlings were cut off directly before freezing in liquid nitrogen. In this case, 30 mg of root material was ground and used for hormone quantification.

### RT-qPCR

Total RNA of Arabidopsis leaves infected with *Botrytis* was extracted after the indicated timepoints using the NucleoSpin RNA Plant kit (Macherey-Nagel, Germany). Reverse transcription of the RNA samples was carried out using the iScript cDNA synthesis kit (Bio-Rad, USA). In a CFX Connect Real-Time PCR Detection System (Bio-Rad, USA) RT-qPCR was performed using the PerfeCTa SYBR Green SuperMix (Quantabio, USA). Actin was used to normalize expression levels and relative expression ratios were calculated according to the efficiency corrected calculation model (Pfaffl 2001). Primer used: Actin_FW: GGGAATGGAAGCTGCTGGAATC, Actin_REV: CCTGGACCTGCCTCATCATACTC; PR1_FW: TTCTTCCCTCGAAAGCTCAA, PR1_REV: AAGGCCCACCAGAGTGTATG.

### GUS staining

GUS staining was performed as described previously (Béziat et al. 2017). In brief: Seven-day-old plate-grown *CYCB1;1::GUS* seedlings were fixed in 90% acetone for 30 min. Following fixation, the seedlings were washed in Na-phosphate buffer for 1 h and then put into freshly prepared staining buffer. After 4 h of staining, the seedlings were transferred into an ethanol:acid mixture to remove chlorophyll. The seedlings were then washed for 10 min each in 70%, 50%, and 20% ethanol and finally transferred into a clearing solution. Images were captured using a Nikon Axiostar Plus microscope and a binocular microscope (Leica M205 FCA).

### Immunolocalization

Whole mount immunolocalization was conducted using 6-day-old Arabidopsis seedlings. Fixation was carried out in 4% formaldehyde solved in microtubule stabilization buffer (MTSB; 25 mm PIPES, 2.5 mm EGTA and 2.5 mm MgSO_4_·7H_2_O; pH 6.8) for 40 min. After washing (MTSB) and rehydration, cell wall digestion (2% driselase in MTSB) and membrane permeabilization (3% NP40 in MTSB) were executed. Subsequent washing was followed by blocking (2%BSA in MTSB) for 2 h and treatment with a 1:300 diluted primary PIN2 antibody (Abas et al. 2006) over night at 4 °C: After extensive washing, a goat anti-rabbit antibody conjugated to PE-CY5.5 (Invitrogen, USA) was applied in 1:400 dilution for minimum 3 h. After final washing and mounting, samples were used for microscopy.

### Immunological detection

For immunological detection, samples were separated via sodium dodecylsulfate polyacrylamide gel electrophoresis (SDS–PAGE). Samples were mixed with loading dye, incubated at 95 °C for 5 min and shortly centrifuged prior to loading. Blotting was performed using either a nitrocellulose (GE Healthcare, USA) or a polyvinylidene difluoride membrane (Sigma-Aldrich). Membranes were blocked with 5% skim milk powder in TBS-T (150 mm NaCl, 10 mm Tris/HCl pH 8.0, 0.1% Tween 20), washed with TBS-T and then incubated with the first antibody solution. After washing the membrane again, it was probed with the second antibody for at least 1 h. Afterwards, the membrane was washed 3 more times, submerged with ECL solution (ECL Prime Kit; Amersham, UK) and chemiluminescence signals were detected using the ChemiDoc system from BioRad. For detection, a monoclonal anti GFP antibody (1:500; Roche) was used. As secondary antibody, horseradish-peroxidase-conjugated goat anti-mouse antibody (1: 5000, Calbiochem) was used.

### 3D surface rendering

3D reconstruction of cells and vacuoles was performed using Imaris 9.1 (Oxford Instruments). For generation of cell models, the manual drawing function (distance) of the surface creation tool was used. Cell borders were marked according to the signals for PI channel on at least every third slice of a z-stack. Subsequently, a surface representing the whole cell was created based on the markings. This surface was used to mask the BCECF-AM channel by setting the voxels outside the surface to 0. For generation of vacuole models, the masked channel was used to automatically create a surface corresponding to the vacuole (BCECF) signals. Before completion, the displayed model was visually compared with the underlying BCECF signals and potential adjustments carried out using the absolute intensity threshold option. Parameters such as sphericity and volume were extracted for individual 3D models. Vacuolar occupancy was determined by dividing the volume of the created 3D vacuole by the total volume of the corresponding cell. This calculation provided the percentage of vacuolar occupation within the cell.

### pH measurements using BCECF

Vacuolar pH of 7-day-old Col-0 seedlings was determined using the fluorescent cell-permeant dye BCECF. Before loading the dye, seedlings were exposed to 50 *µ*M SA in liquid ½ MS + medium for varying durations or grown on SA plates for 7 days. For staining, 10 *µ*M BCECF-AM was added to the SA-supplemented liquid medium, and the seedlings were incubated in the dark for 1 h. Following staining, the seedlings were washed for 10 min in fresh liquid ½ MS + medium. For microscopy, the fluorophore was excited at 488 and 458 nm, respectively, and the emission was detected between 530 and 550 nm. The ratio of fluorescence intensity was determined using ImageJ by dividing the values of the 488-nm-excited images by those of the 458-nm-excited images.

### V-ATPase activity assay

For measuring the activity of the vacuolar ATPase seedlings were grown on solid ½MS + medium and after 4 days transferred to liquid ½MS + medium for 2 wk. The seedlings were treated with 50 *µ*M SA for 5 h, or with DMSO as control. The roots were isolated and flashed frozen in liquid nitrogen. Microsomal fractions were collected from total protein extracts by ultracentrifugation. The microsomes were resuspended in homogenization buffer (50 mm Tris-HCl, pH 7.5; 150 mm NaCl; 10% glycerol; 2 mm EDTA; 5 mm DTT; 1% protease inhibitor cocktail; 1% IGEPAL; 1 mm NaF; 1 mm PMSF) to a protein concentration of 0.45 *µ*g/µL. Then the ATP hydrolytic activity of NaNO₃-sensitive V-ATPases were measured by quantifying phosphate-malachite complexes as a proxy for the released inorganic phosphate.

### Statistical analysis

All quantitative data was analyzed using the GraphPad Prism 9 software. The precise statistical method used are given in the respective figure legends.

### Accession numbers

Sequence data from this article can be found in the GenBank/EMBL data libraries under accession numbers At5g57090 (PIN2), At1g25490 (PP2A), At5g16830 (SYP21), At1g64280 (NPR1), At5g45110 (NPR3), At4g19660 (NPR4), At1g03470 (NET3C), At5g58320 (NET4A), At2g30500 (NET4B), At2g21410 (VHA-A2), At4g39080 (VHA-A3).

## Supplementary Material

kiaf439_Supplementary_Data

## Data Availability

The data underlying this article will be shared on reasonable request to the corresponding author.

## Supplementary data

The following materials are available in the online version of this article.

**Supplementary Figure S1.**  *Botrytis* infection induces phytohormone accumulation.

**Supplementary Figure S2.** SA reduces root organ growth independently of the NPR receptors.

**Supplementary Figure S3.** SA treatment impacts cell division, leading to altered meristem size.

**Supplementary Figure S4.** The functional SA analogs INA and BTH do not show SA-related phenotypes.

**Supplementary Figure S5.** Changes in vacuolar occupancy upon prolonged auxin or SA treatment.

**Supplementary Figure S6.** Calibration curve for determining absolute vacuolar pH.

**Supplementary Figure S7.** Root growth quantification and vacuolar pH measurements of *UGT76B1* overexpression and knockout lines upon SA treatment.

**Supplementary Video S1.** Application of SA changes vacuolar morphology within minutes.

**Supplementary Video S2.** SA promotes homotypic vacuole fusion.
