# Supplementary material for: Salicylic acid inhibits V-ATPase activity and restricts cell elongation
Source: Plant Physiol. 2025 Sep 26;199(2):kiaf439. doi: 10.1093/plphys/kiaf439 (PMC12532109; doi:10.1093/plphys/kiaf439)
Supplement: kiaf439_Supplementary_Data [file kiaf439_supplementary_data.zip › PLPHYS-2025-0492R1_Supplementary Video Legends.pdf]

Supplementary Video Legends

Supplementary Video S1. Application of SA changes vacuolar morphology within minutes. 50 µM SA were applied to Arabidopsis seedlings expressing the tonoplast marker YFP-VAMP711. The video captures root growth from minute 5-50 after SA application.

Supplementary Video S2. SA promotes homotypic vacuole fusion.
50 µM SA were applied to Arabidopsis seedlings expressing the tonoplast marker YFP-VAMP711. The video captures root growth from minute 50-180 after SA application.
