# Supplementary material for: Salicylic acid inhibits V-ATPase activity and restricts cell elongation
Source: Plant Physiol. 2025 Sep 26;199(2):kiaf439. doi: 10.1093/plphys/kiaf439 (PMC12532109; doi:10.1093/plphys/kiaf439)
Supplement: kiaf439_Supplementary_Data [file kiaf439_supplementary_data.zip › renamed_12be7.pdf]

Supplemental Information

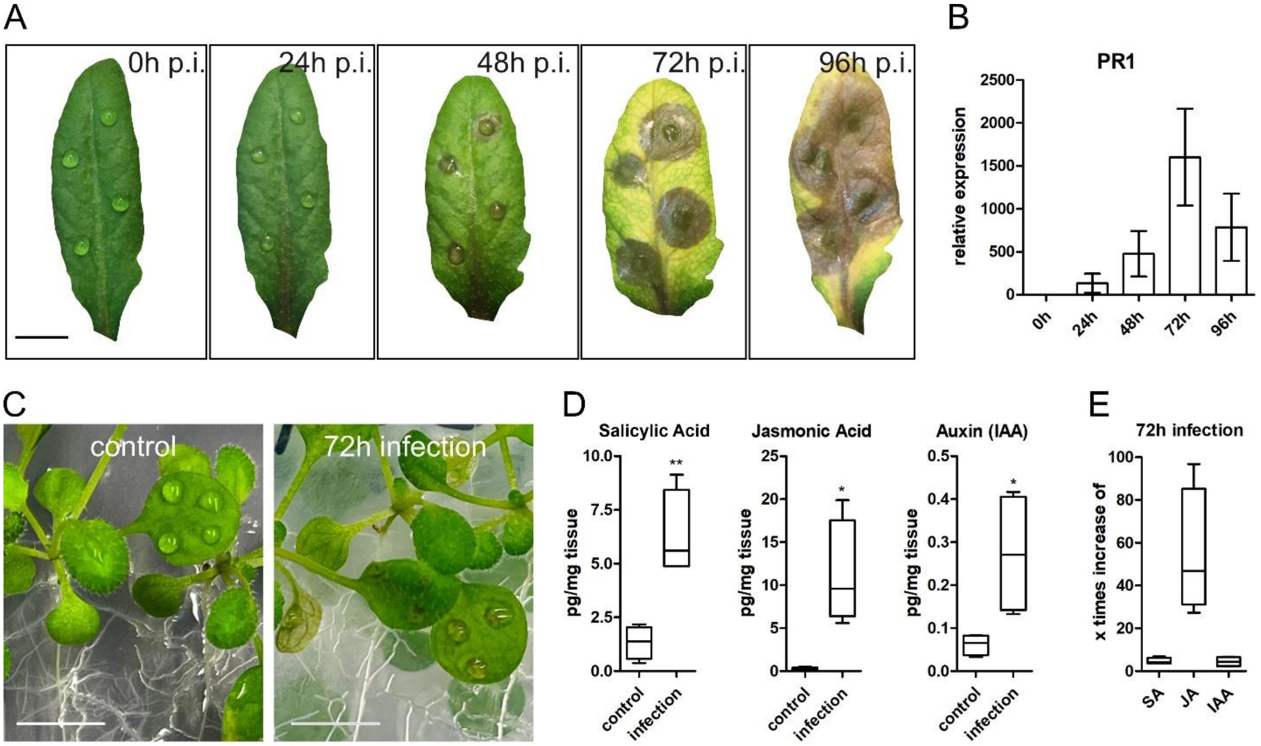

Supplementary Figure S1. *Botrytis* infection induces phytohormone accumulation. **A)** *Botrytis cinerea* infection on adult Arabidopsis leaves over a period of 96 hours. Pictures taken after 24h, 48h, 72h, and 96h. Images were digitally extracted for comparison. 5µl spore suspension with $5{\times}10^4$ spores/mL in Gamborg medium containing 25 mM glucose was applied. Scale bar: 1 cm. **B)** RT-qPCR analysis of the SA marker gene *PR1* after 24h, 48h, 72h, and 96h of infection, normalized to the 0h time point. Bar graph shows the mean of four biological replicates with the standard error. **C,D)** Arabidopsis seedlings were grown for 2 weeks on ½MS+ medium, inoculated with 1 µl spore suspension of $5{\times}10^4$ spores/mL in Gamborg medium containing 25 mM glucose, and incubated for 72h. Whole seedlings (60 mg) were ground, and the levels of salicylic acid (SA), jasmonic acid (JA), and auxin (IAA) were measured (n control = 4, n 50 µM SA = 4). Scale bar: 0.5 cm. Data are presented in a whisker plot with Student's t-test. $^*P \leq 0.05$, $^{**}P \leq 0.01$. **E)** Fold increase of phytohormone levels upon Botrytis infection normalized to the mock infected samples (control). Box limits in the graphs represent 25th–75th percentile, the horizontal line the median and whiskers minimum to maximum values.

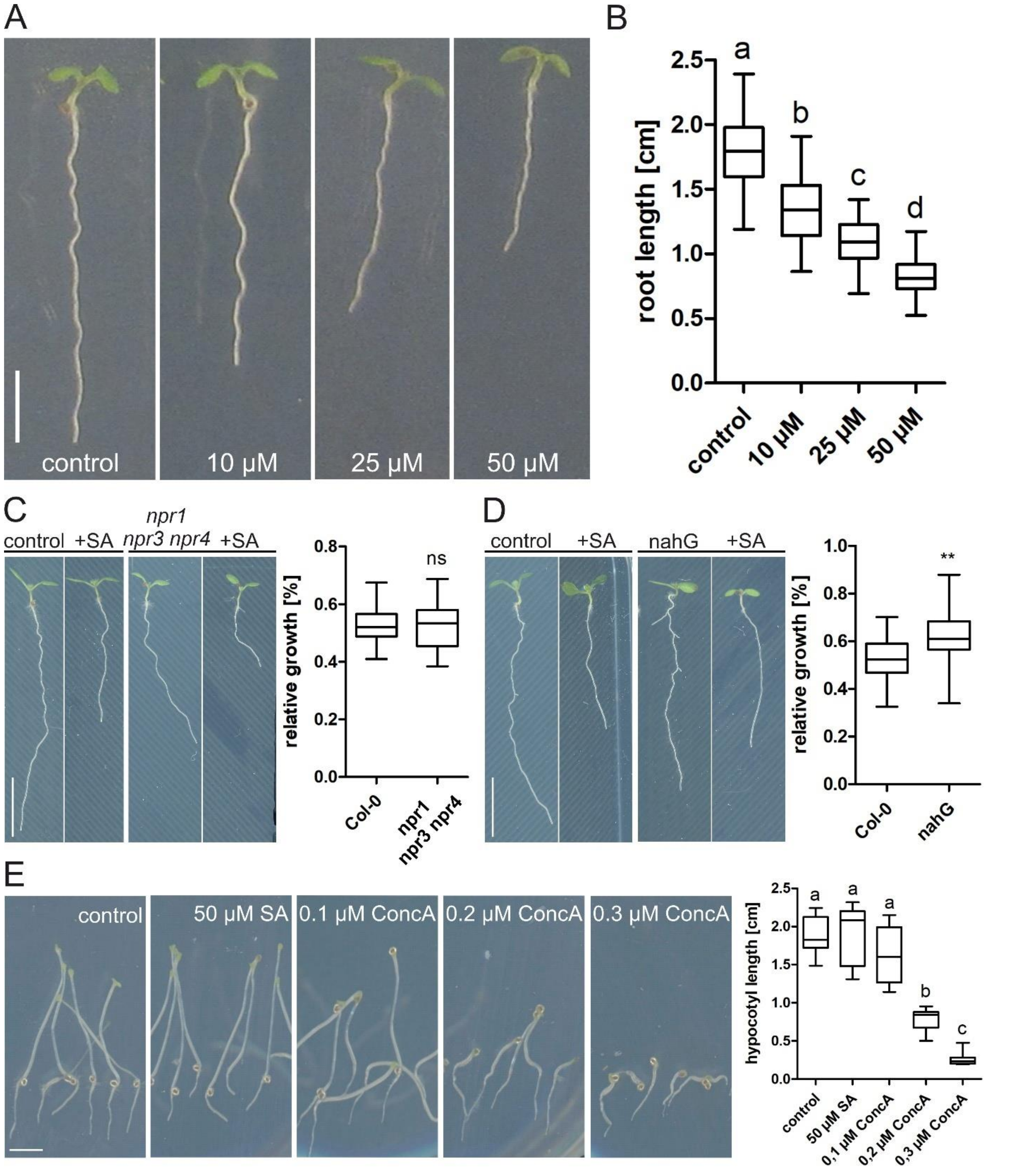

Supplementary Figure S2. SA reduces root organ growth independently of the NPR-receptors. **A)** Representative images showing root length of Col-0 grown for 7 days on different concentrations of SA. The images for “control” and “50 µM” are the same than shown in Figure 1C. Scale bar applies to all images in A): 40 mm. Note: values for control and 50 µM SA are included in the graph for Fig 1D. **B)** Quantitative analysis of Col-0 root length grown for 7 days on different concentrations of SA. Data is presented in a whisker plot. Statistical analysis was performed using a one-way ANOVA test with Tukey post-hoc test. Change in letter equals $P \leq 0.001$. **C-D)** Col-0, *npr1 npr3 npr4*, and nahG seedlings were grown for 7 days on ½MS+ plates supplemented with 50 µM SA. Calculation of relative growth: root length of seedlings grown on SA is divided by mean root length of seedlings from control plates (n *npr1 npr3 npr4* = 21, n nahG = 20).

Scale bar applies to all images in C) and D): 40 mm. Data is presented in a whisker plot with Student's t-test. ***P* ≤ 0.01. **E)** Col-0 seeds were placed on plates containing different concentrations of Concanamycin A (ConcA) and kept in light for 8 hours before being transferred to the dark for an additional 5 days. Hypocotyl length was measured after 5 days of incubation in the dark (n control = 9, n 50 μM SA = 10, n 0.1 μM ConcA = 10, n 0.2 μM ConcA = 9, n 0.3 μM ConcA = 8). Scale bar applies to all images in E): 0.5 cm. Data are presented in a whisker plot. Statistical analysis was performed using a one-way ANOVA test with Tukey post-hoc test. Change in letter equals *P* ≤ 0.001. Box limits in whisker plots represent 25th–75th percentile, the horizontal line the median and the whiskers minimum to maximum values.

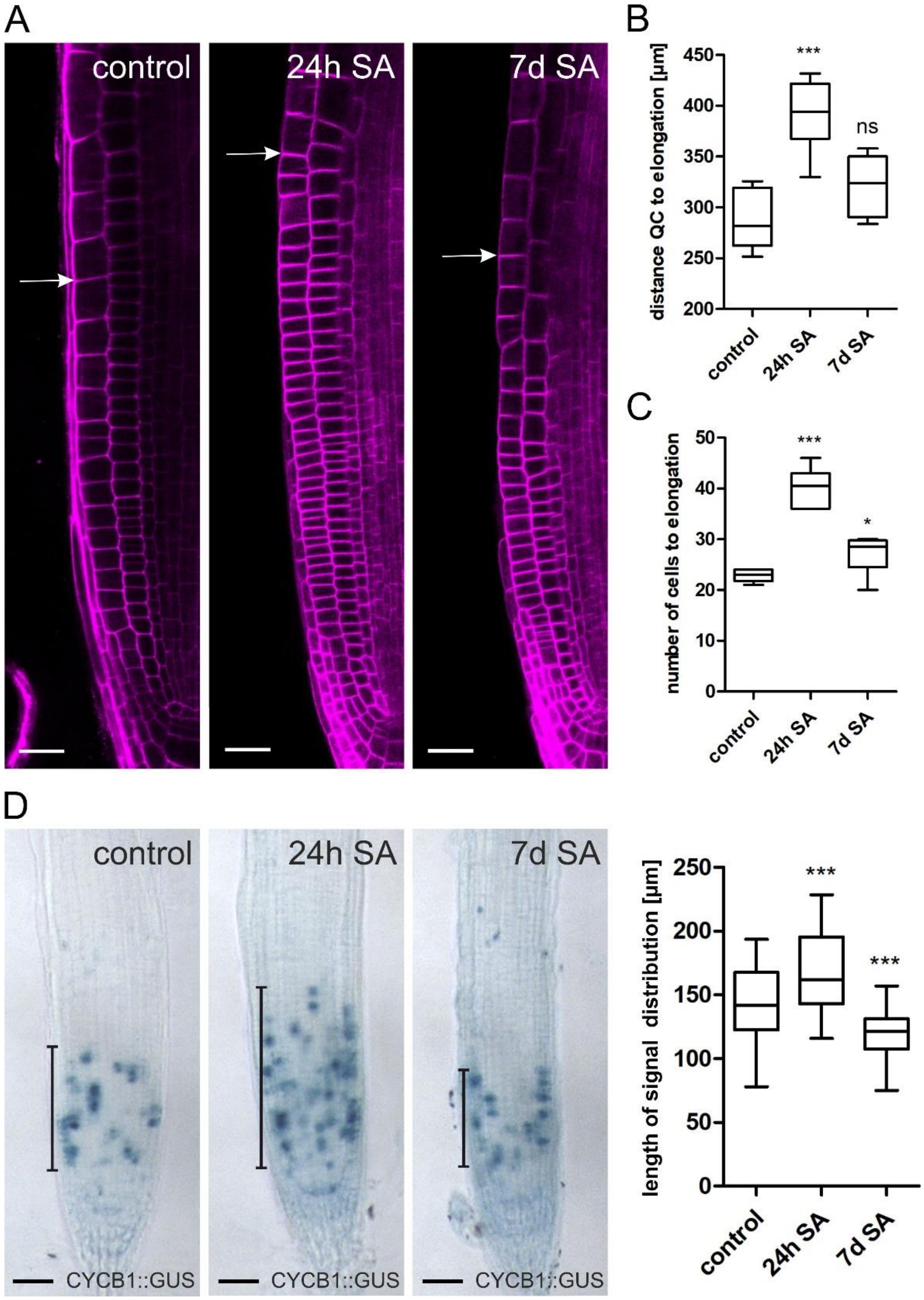

Supplementary Figure S3. SA treatment impacts cell division, leading to altered meristem size. **A)** Root cross-section stained with PI. The onset of elongation, defined as the first cell being longer than wide, is indicated (white arrow). Scale bar: 30 µm. **B)** Quantification of the distance from the quiescent centre (QC) to the first elongating cell (n control = 5, n 24h SA = 6, n 7d SA = 8). Data is presented in a whisker plot with one-way ANOVA and Tukey post-hoc test. ***$P \leq 0.001$, ns = not significant **C)** Count of cells between the QC and the first elongating cell (n control = 6, n 24h SA = 6, n 7d SA = 8). Data are presented in a whisker plot with one-way ANOVA and Tukey post-hoc test. ***$P \leq 0.001$, *$P \leq 0.05$. **D)** The marker for mitotic activity, cyclin B fused to glucuronidase (CYCB1::GUS), was used to follow cell division in the meristem

after 24h and 7d SA (50 µM) treatment. The length of signal distribution, indicated by black bars, was quantified (n control = 35, n 24h SA = 31, n 7d SA = 39). Scale bar: 0.8 mm. Data is presented in a whisker plot with one-way ANOVA and Tukey post-hoc test. ***$P \leq 0.001$. Box limits in all whisker plots represent 25th–75th percentile, the horizontal line the median and the whiskers minimum to maximum values.

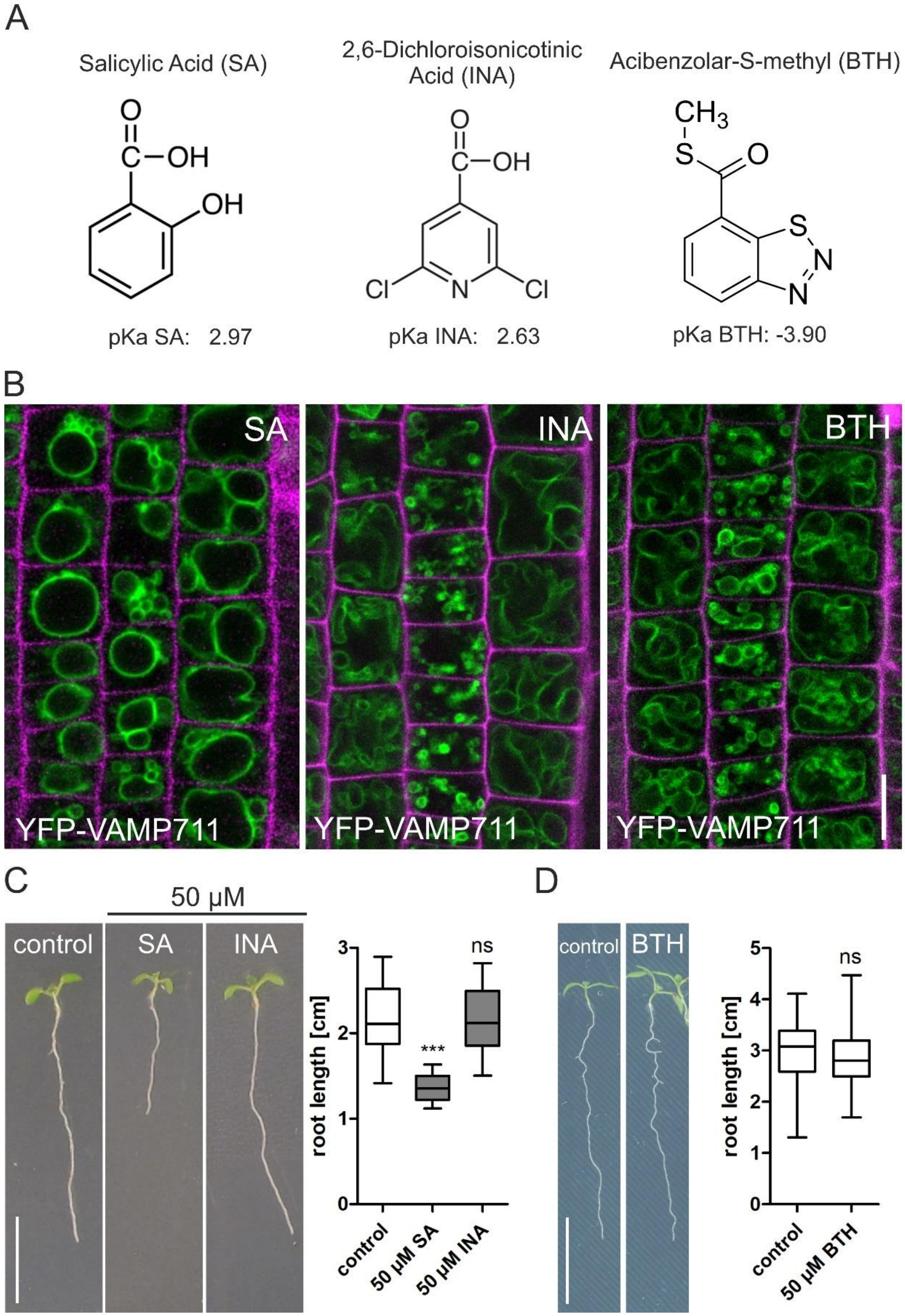

Supplementary Figure S4. The functional SA analogs INA and BTH do not show SA-related phenotypes. **A, B)** Structural formula and pKa value of SA, 2,6-dichloronicotinic acid (INA), and acibenzolar-S-methyl (BTH). The tonoplast marker line pUBQ10::YFP-VAMP711 was used to visualize vacuole changes after 24h treatment with 50 µM SA, INA, or BTH. Scale bar: 15 µm. **C)** Root growth determination: Col-0 seedlings were grown for 7 days on ½MS+ plates supplemented with 50 µM SA or INA in comparison to the untreated

control. The relative growth was calculated by dividing each root length of seedlings grown on SA- or INA-supplemented media by the average root length of their respective controls (n control = 20, n SA = 22, n INA = 22). Scale bar: 1 cm. Data is presented in a whisker plot with Student's t-test. ***$P \leq 0.001$. **D)** Col-0 seedlings were grown for 7 days on ½MS+ plates supplemented with 50 µM BTH in comparison to the untreated control (n control = 116, n BTH = 110). Scale bar: 1.4 cm. Data is presented in a whisker plot with Student's t-test. ns = not significant. Box limits in all whisker plots represent 25th–75th percentile, the horizontal line the median and the whiskers minimum to maximum values. Scale bars always apply to the entire set of neighboring pictures.

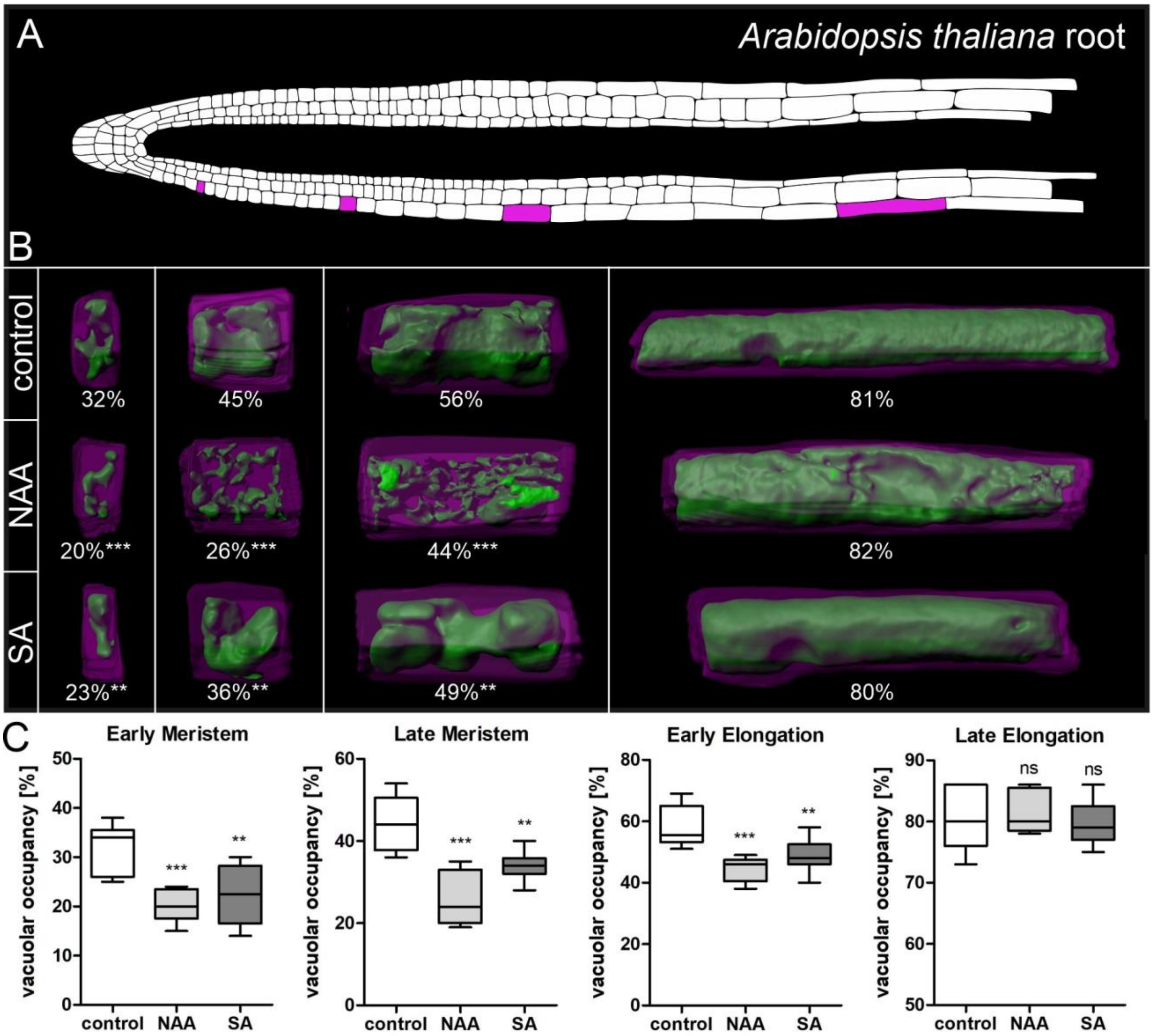

Supplementary Figure S5. Changes in vacuolar occupancy upon prolonged auxin or SA treatment. **A)** Schematic representation of an Arabidopsis thaliana root showing epidermis, cortex, and endodermis cell files. The investigated region is highlighted in magenta. **B)** Col-0 seedlings were grown for 7 days on ½MS+ medium supplemented with 50 µM SA and 250 nM NAA. Seedlings were stained with PI and BCECF, and Z-stacks were acquired using CLSM. From these, 3D reconstructions of the vacuole (green) and its corresponding cell (magenta) were generated, and vacuolar occupancy was quantified. The columns represent the following regions from left to right: early meristem, late meristem, early elongation, and late elongation zones. The numbers under each image indicate the mean percentage of vacuolar occupancy. For comparison, student's t-tests were carried out (control = vacuoles from untreated seedlings): $^{**}P \leq 0.01$, $^{***}P \leq 0.001$. **C)** Quantification of vacuolar occupancy across the four root regions (n = 10). Data is presented in a whisker plot with Student's t-test. $^{**}P \leq 0.01$, $^{***}P \leq 0.001$, ns = not significant. Box limits represent 25th–75th percentile, the horizontal line the median and the whiskers minimum to maximum values.

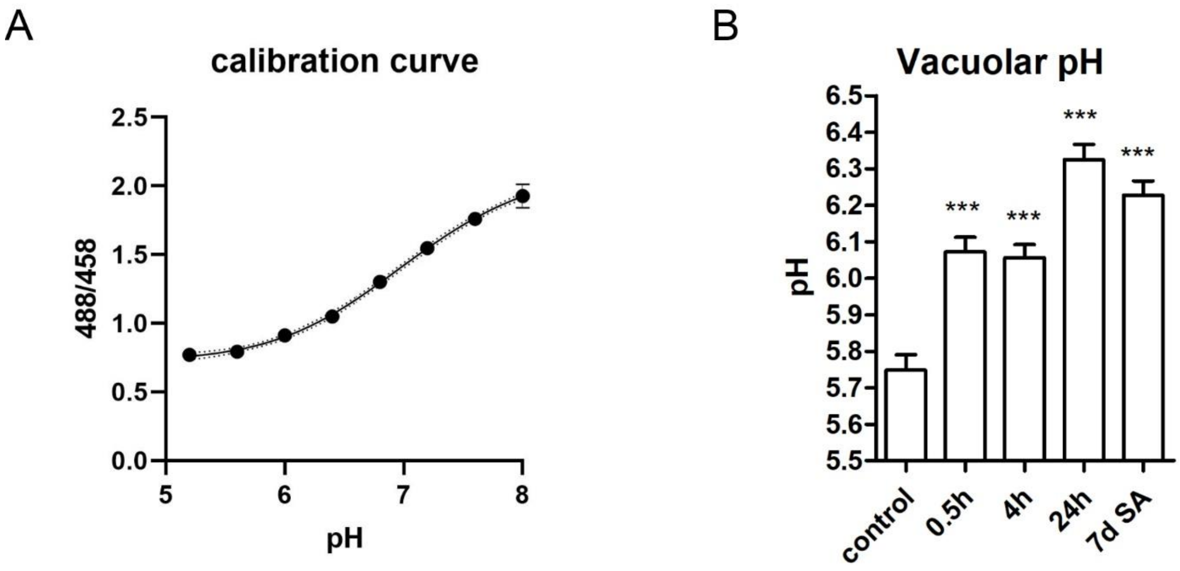

Supplementary Figure S6. Calibration curve for determining absolute vacuolar pH. **A)** A calibration curve was created by staining 7-day-old seedlings with BCECF and submerging them for 10 minutes in a sequence of different pH solutions (pH 5.2, 5.6, 6.0, 6.4, 6.8, 7.2, 7.6, and 8.0). The λex 488 value was divided by λex 458, and a sigmoidal curve was fitted to the resulting data. **B)** Quantification of relative vacuolar pH was performed across all root regions combined, using BCECF to normalize the λex 488/λex 458 ratio against the DMSO control (n = 30). Data was analyzed using one-way ANOVA with Tukey post-hoc test. ****P* ≤ 0.001. Means are shown with standard error.

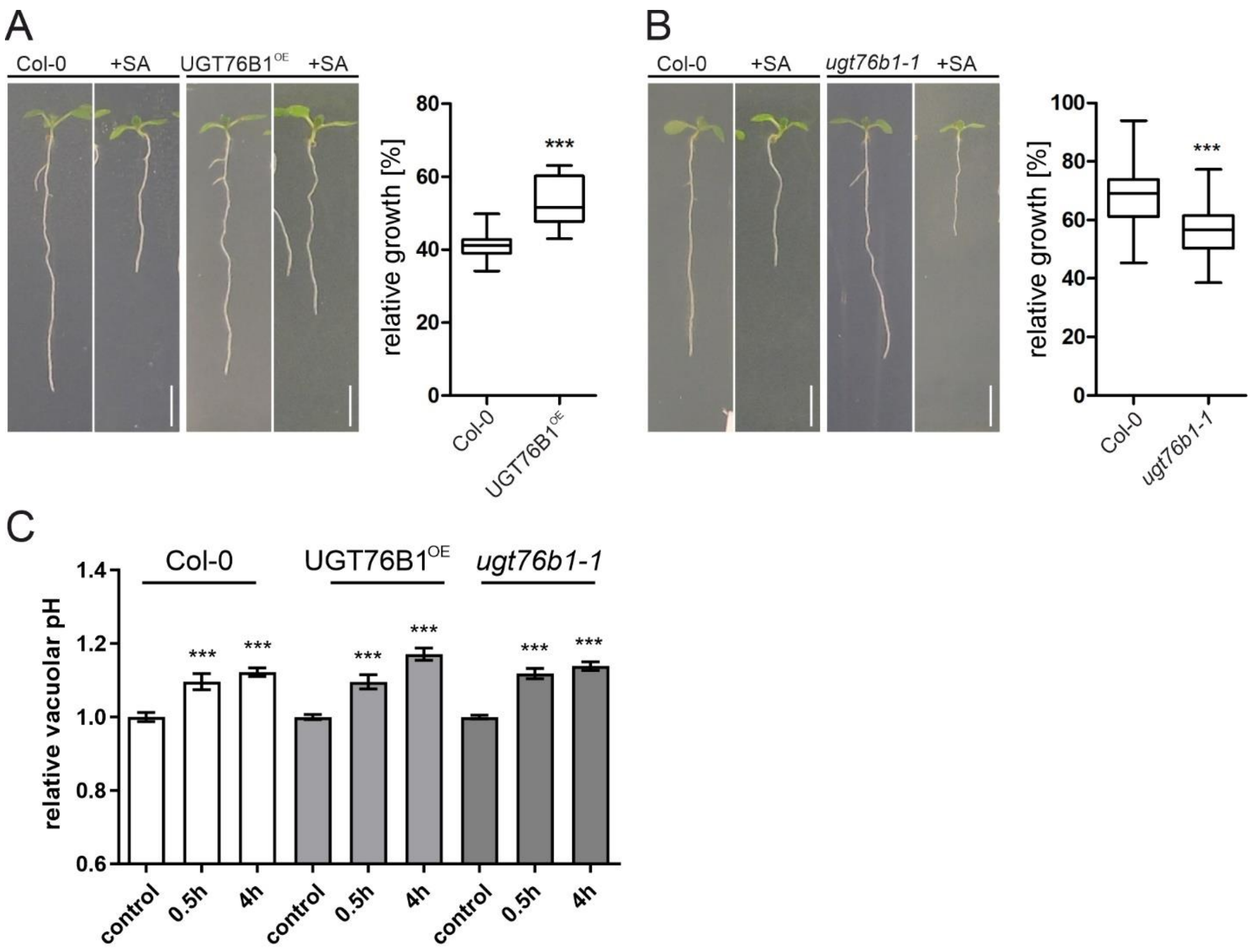

Supplementary Figure S7. Root growth quantification and vacuolar pH measurements of UGT76B1 overexpression and knockout lines upon SA treatment. **A, B)** Col-0, UGT76B1^{OE}, and *ugt76b1-1* seedlings were grown for 7 days on ½MS+ plates supplemented with 50 µM SA. Relative growth was calculated by dividing the root length of seedlings grown on SA-supplemented media by the average root length of their respective controls (n Col-0 = 17, n UGT76B1^{OE} = 19 for A; n Col-0 = 43, n *ugt76b1-1* = 42 for B). Scale bars also always apply to the control picture to the left: 30 mm. Data are presented in a whisker plot with Student's t-test. ****P* ≤ 0.001. Box limits represent 25th–75th percentile, the horizontal line the median and the whiskers minimum to maximum values. **C)** Quantification of relative vacuolar pH was performed using BCECF by normalizing the λex 488/λex 458 ratio to the DMSO control (n Col- 0 control = 25, n Col-0 0.5h = 14, n Col-0 4h = 22, n UGT76B1OE control = 25, n UGT76B1^{OE} 0.5h = 14, n UGT76B1^{OE} 4h = 22, n *ugt76b1-1* control = 25, n *ugt76b1-1* 0.5h = 14, n *ugt76b1-1* 4h = 22). Data was analyzed using ANOVA with Tukey post-hoc test. ****P* ≤ 0.001. Means are shown with standard error.
